# Supramolecular Amino Acid Based Hydrogels: Probing the Contribution of Additive Molecules using NMR Spectroscopy

**DOI:** 10.1002/chem.201700793

**Published:** 2017-05-23

**Authors:** Susana M. Ramalhete, Karol P. Nartowski, Nichola Sarathchandra, Jamie S. Foster, Andrew N. Round, Jesús Angulo, Gareth O. Lloyd, Yaroslav Z. Khimyak

**Affiliations:** ^1^ School of Pharmacy University of East Anglia Norwich Research Park NR4 7TJ UK; ^2^ Institute of Chemical Sciences, School of Engineering and Physical Sciences Heriot-Watt University EH14 4AS UK; ^3^ Current address: Department of Drug Form Technology Faculty of Pharmacy Wroclaw Medical University ul. Borowska 211 50-556 Wroclaw Poland

**Keywords:** additive molecules, NMR spectroscopy, self-assembly, STD NMR, supramolecular chemistry

## Abstract

Supramolecular hydrogels are composed of self‐assembled solid networks that restrict the flow of water. l‐Phenylalanine is the smallest molecule reported to date to form gel networks in water, and it is of particular interest due to its crystalline gel state. Single and multi‐component hydrogels of l‐phenylalanine are used herein as model materials to develop an NMR‐based analytical approach to gain insight into the mechanisms of supramolecular gelation. Structure and composition of the gel fibres were probed using PXRD, solid‐state NMR experiments and microscopic techniques. Solution‐state NMR studies probed the properties of free gelator molecules in an equilibrium with bound molecules. The dynamics of exchange at the gel/solution interfaces was investigated further using high‐resolution magic angle spinning (HR‐MAS) and saturation transfer difference (STD) NMR experiments. This approach allowed the identification of which additive molecules contributed in modifying the material properties.

## Introduction

Hydrogels are semi‐solid colloidal materials which contain a three‐dimensional (3D) network with a fine structure that can often be defined by non‐covalent interactions.^1^, ^2^ They contain large amounts of water, entrapped by capillary and surface forces,^1^, ^2^ which provide liquid‐like properties to these solid‐like rheological systems.^3^, ^4^ This feature explains their great resemblance to human tissues.^3^ In combination with the use of biocompatible and biodegradable precursors, hydrogels have a recognised potential as matrices for cell and tissue engineering, scaffolds for wound healing and vehicles for drug delivery.^3^ Their use in targeted and controlled drug delivery is based on their responsiveness to the environment,^5^ therefore receiving the designation of *Smart Materials*.^6^ Physiological stimuli, such as temperature, pH and/or biological compounds may trigger changes in the structural and physicochemical properties of smart hydrogels leading to drug release, degradation or dissolution of the 3D network, induction of phase transitions and/or alteration of shape.^5^

Low molecular weight gelators (LMWG), with a molecular mass normally under 1000 Da, are molecules capable of self‐assembling into organised gel networks.^7^ Amino acids are naturally abundant molecules that show great potential for hydrogelation due to their amphiphilic character; the hydrophilic moieties create opportunities for hydrogen bonding and stabilisation of water molecules, whereas the hydrophobic moieties may prompt aggregation.^8^ Weak interactions between complimentary amino acids occur in nature and their importance has been demonstrated in many biological processes, such as protein folding. These essential building blocks of life have their function dictated by the type of amino acids and interactions participating in their stabilisation.^7^, ^9^, ^10^, ^11^

**Figure 1:**
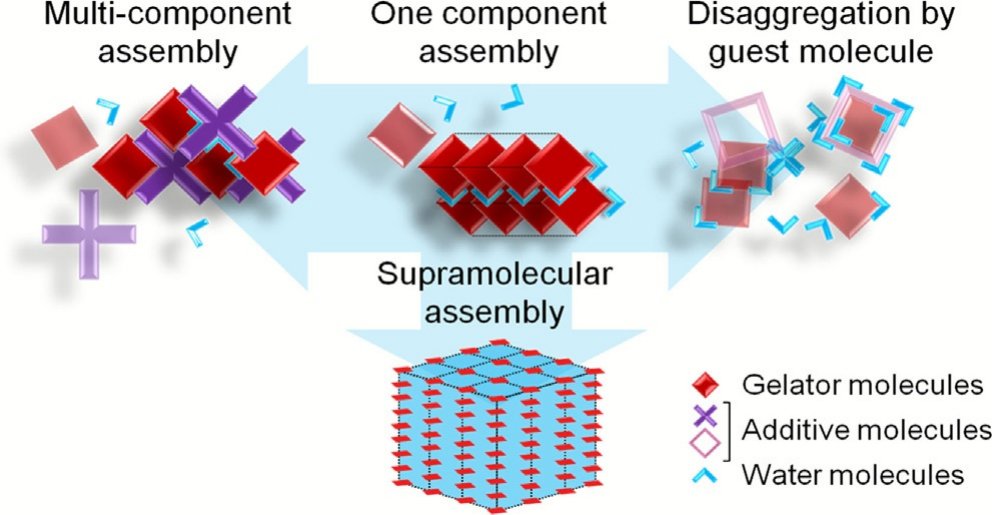
LMWG form complex supramolecular structures through non‐covalent interactions (mainly H‐bonding, van der Waals interactions and/or π–π stacking). The anisotropy of the interactions favours their self‐assembly in one dimension and leads to the formation of fibres, which grow, entangle and create bundles, ultimately leading to the formation of a 3D network (bottom).^15^ The introduction of additive molecules was investigated regarding their effects on the organisation of the 3D network. They can either modify the physical properties of the hydrogel (left) or prevent its formation (right).

To date, l‐phenylalanine (Phe) is the smallest molecule known to form supramolecular hydrogels.^12^ Hydrogelation of Phe was first described in the literature in 2002 by Myerson et al. as producing a hydrogel containing crystals (the material was named a gel‐crystal).^12^ The importance of investigating hydrogelation of Phe was raised recently when several studies correlated the aggregation of Phe with phenylketonuria and β‐amyloid‐based neurodegenerative conditions, such as Alzheimer's and Huntington's diseases.^10^, ^13^ Considering the pathological implications of aggregation of Phe and the potential applications of Phe‐based hydrogels as drug delivery matrices, we developed amino acid based multi‐component gelating systems. The introduction of co‐gelators or non‐gelating additive molecules has been reported previously in other gel systems to create an additional level of control and therefore to allow tailoring of the physical properties of gels through modifications to their supramolecular structure (Figure 1).^14^

**Figure 2:**
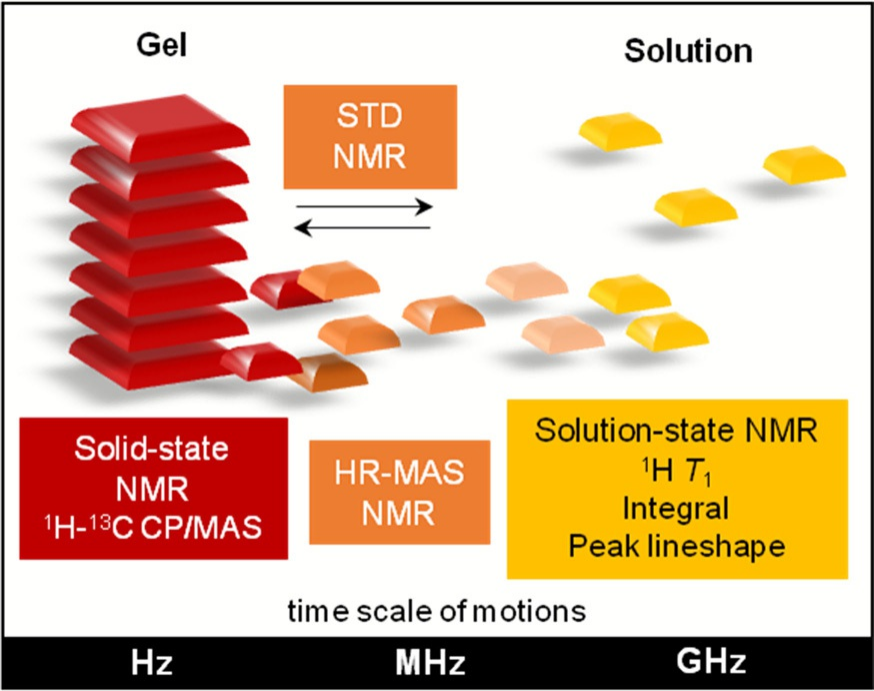
Schematic representation of the application of NMR methods to probe different time scales of motions and self‐organisation in supramolecular materials at different length scales. While the rigid fibres can be studied by ^1^H‐^13^C CP/MAS solid‐state NMR experiments, structure and dynamics of mobile molecules were characterised by spectral variations and ^1^H *T*_1_ measurements from solution‐state NMR experiments. Molecules at gel/solution interfaces with intermediate frequencies of motion can be assessed using HR‐MAS NMR experiments, and their dynamics of exchange studied by solution‐state STD NMR.

**Figure 3:**
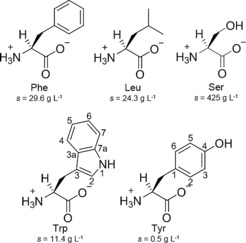
Zwitterionic structures of the amino acids in study with the corresponding solubilities in water at 298 K (Phe: l‐phenylalanine, Leu: l‐leucine, Ser: l‐serine, Trp: l‐tryptophan and Tyr: l‐tyrosine). Trp and Tyr structures are numbered.

Despite the wide range of existing and potential applications of hydrogels,^2^, ^3^, ^16^ fundamental understanding of the mechanism of formation of their complex fibrous networks is still very limited. Such knowledge is important to design and control the properties of these materials for specific applications, and to get a molecular level insight into the pathological aggregation processes of certain amino acids. Therefore, the main purpose of this work was to gain a molecular level understanding of Phe‐based hydrogels, used as model multiphasic materials, through the application of complementary methods, including rheology, microscopy, diffraction and advanced nuclear magnetic resonance (NMR) spectroscopy (Figure 2). Cross‐polarisation solid‐state NMR experiments were used to detect the solid‐like fibrous components, whereas pulsed‐field gradient high‐resolution magic angle spinning (HR‐MAS) NMR experiments were performed in order to investigate the fibre/solution interfaces. Furthermore, characterisation of spectral variations from solution‐state NMR experiments and measurement of their relaxation times throughout the gel‐to‐solution transitions were carried out to describe structure and dynamics of species in solution. Finally, saturation transfer difference (STD) NMR experiments were applied to assess exchange phenomena at the gel/solution interfaces, with particular potential for the identification of the role of each amino acid in the processes of self‐assembly of multi‐component materials. Only the combination of solution, solid‐state and HR‐MAS NMR experiments has enabled us to gain in‐depth understanding of such complex, multiphasic soft materials. To further explore the potential uses of Phe‐based hydrogels, we introduced potential co‐gelators or non‐gelating additive molecules in the form of related hydrophilic and hydrophobic amino acids (Figure 3).

## Results

### Macroscopic observations and fibre morphology determination

**Figure 4:**
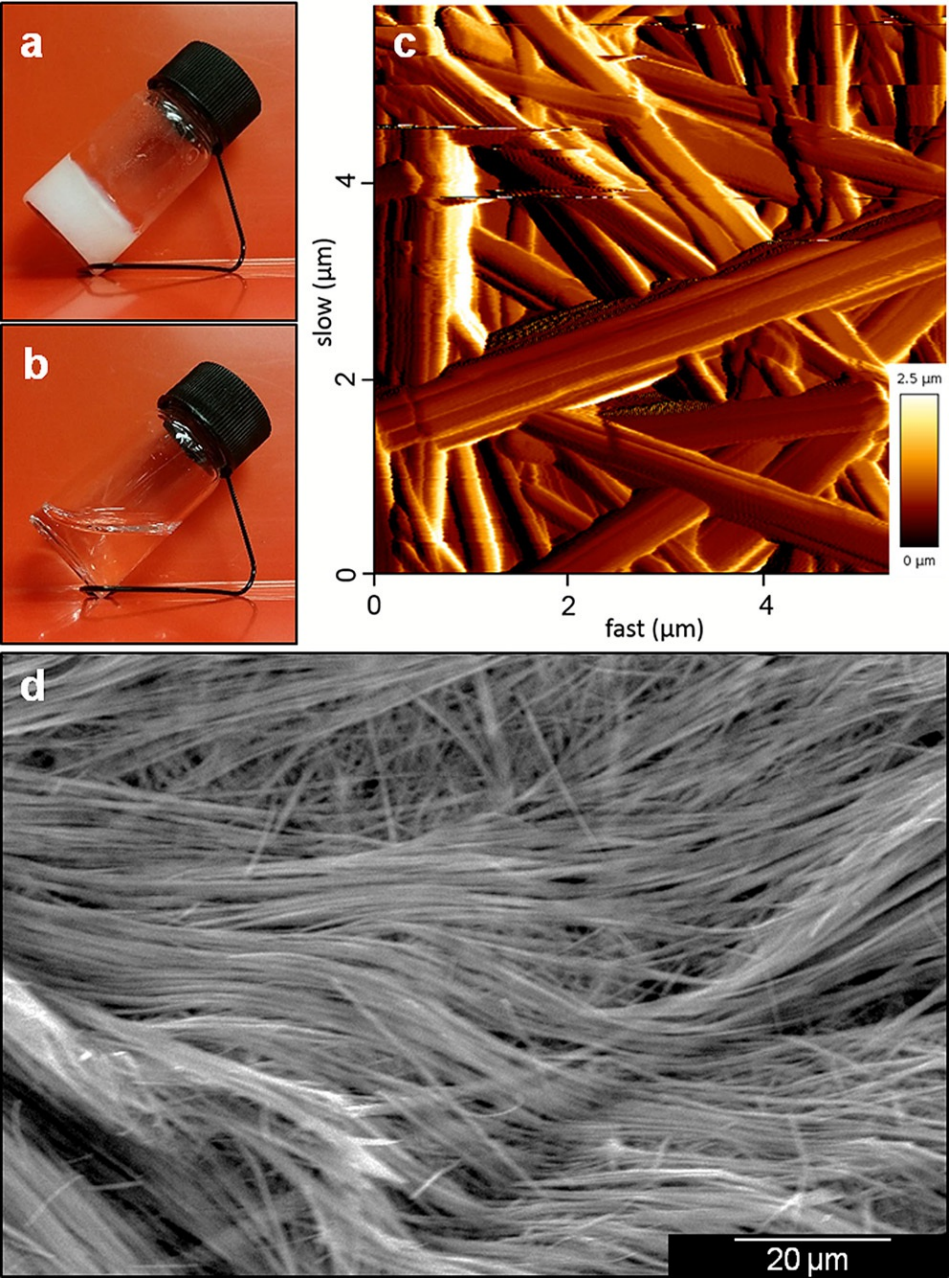
Thermoreversible hydrogel of Phe (303 mm) a) below and b) above *T*_gel_. c) AFM and d) SEM images of dried hydrogel of Phe (303 mm), highlighting the entanglement of a fibrous network with fibre widths ranging from 200 to 900 nm.

Solutions of Phe have shown thermoreversible formation of hydrogels that sustain their own weight under inversion in the range of concentrations from 212 to 605 mm. Throughout the gel forming concentrations, 303 mm Phe represented the rheologically strongest material.^17^ During the process of cooling down, the formation of white cloud‐like centres was observed, followed by growth and entanglement of fibres, ultimately leading to the formation of a white opaque hydrogel (Figure 4 a). Microscopic analysis of the hydrogels has shown the presence of thin fibres with an average width of 440 nm (Figure 4 c and d, and Figure S1 in Supporting Information). Fast quenching of the solutions was essential to ensure gelation, as gradual lowering of temperature led to the formation of solid precipitates.

The hydrogelation ability of a range of structurally diverse amino acids with different hydrophilicities (Figure 3) was also tested. Suspensions of l‐leucine (Leu), l‐serine (Ser), l‐tryptophan (Trp) and l‐tyrosine (Tyr) formed clear solutions when heated. When subjected to the same cooling conditions, all formed crystalline precipitates in the range of 5 to 500 mm. Suspensions of Phe mixed with these non‐gelating additives (total concentration of 303 mm) were heated and quenched, resulting in white opaque hydrogels obtained from the mixtures Phe/Leu (5:1), Phe/Ser (5:1), Phe/Trp (5:1) and Phe/Tyr (5:1). The pH values for the pure sample and mixtures were not significantly different (Table S1 in Supporting Information).

The temperature above which supramolecular arrangements were broken (i.e., loss of structural integrity reflected by the ability to flow under inversion) was defined as the gel‐to‐solution transition (*T*_gel_). *T*_gel_ values for hydrogels of Phe were not affected upon the introduction of these additive molecules. These temperatures were in the range of 322 to 327 K for all the hydrogels (Table S2 in Supporting Information).

### Resistance of hydrogels to deformation

Deformation of hydrogel fibres under stress was studied through the determination of their viscoelastic parameters. During frequency sweep studies, the angle formed between the phases of stress and strain, defined as the phase angle (δ), can range from 0°, for ideal solids, to 90°, for Newtonian liquids.^11^ When frequency sweeps were performed with a small amplitude stress, a phase angle of ca. 10° was recorded; an indication of these hydrogels’ solid‐like nature.

**Figure 5:**
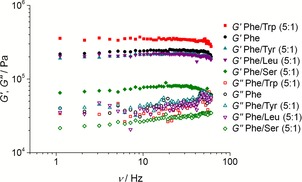
Storage (*G′*) and loss (*G′′*) moduli at increasing frequency sweeps for the hydrogels of Phe/Trp (5:1), Phe, Phe/Tyr (5:1), Phe/Leu (5:1) and Phe/Ser (5:1). Measurements of storage and loss moduli of supramolecular hydrogels were reproducible within an error of 10.7 %.

The storage modulus (*G*′) reflects the elasticity of a material, while the loss modulus (*G*′′) shows the amount of energy lost from the system by non‐elastic behaviour.^11^ *G*′ values for the majority of these hydrogels were in the order of 10^5^ Pa (*G*′≈2.0×10^5^ Pa) and were typically two to five times greater than the *G*′′ values (*G*′′≈4.5×10^4^ Pa) (Figure 5), a relationship characteristic of robust gels, which demonstrates the elastic behaviour of these materials.^11^

Similar *G*′ values were obtained for the hydrogels of Phe, Phe/Leu and Phe/Tyr (*G*′≈2.0×10^5^ Pa), indicating that the presence of Tyr or Leu did not modify significantly the response of the hydrogel fibres to stress. However, significant differences between these systems and the hydrogels of Phe/Trp (*G*′=3.5×10^5^ Pa) and Phe/Ser (*G*′=7.5×10^4^ Pa) were identified. The addition of Trp increased the storage modulus, indicative of a rheologically stronger network. In contrast to Trp, the addition of Ser severely decreased the resistance of the hydrogel to deformation.

### Structural characterisation of the fibrous network

**Figure 6:**
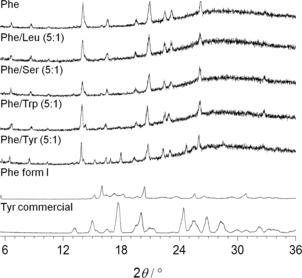
PXRD patterns of the hydrogels of Phe, Phe/Leu (5:1), Phe/Ser (5:1), Phe/Trp (5:1) and Phe/Tyr (5:1) and reference solid powders of the anhydrous form I of Phe and the commercially available Tyr (CSD ref. LTYROS02).^20^ Complete description of diffraction *d*‐spacings can be found in the Supporting Information (Figure S4).

The crystalline nature of the hydrogel fibres of Phe was revealed by the presence of diffraction peaks in PXRD patterns of single and multi‐component hydrogels (Figure 6). All patterns exhibited a broad low intensity hump centred at 28° 2*θ*, assigned to water molecules.^18^ When dried, this “halo” peak disappeared.

Hydrogels of Phe can self‐organise into the monohydrate phase of Phe in the range of 212 to 303 mm.^17^ Very similar diffraction patterns were recorded for multi‐component hydrogels, indicating the presence of the Phe monohydrate phase as the building block of the hydrogel fibres, despite the introduction of additive molecules. Additional diffraction peaks were identified in the PXRD patterns of Phe/Tyr gels (Figure 6), attributed to needle‐like crystals of Tyr immersed in a very dense fibrous network. These were also detected by polarised light microscopy and SEM (Figure S3 in Supporting Information). Despite Tyr being poorly soluble in water (0.451 g L^−1^ at 298 K),^19^ which prevented its full dissolution, the same concentrations were used to match the studies performed with the other amino acids.

**Figure 7:**
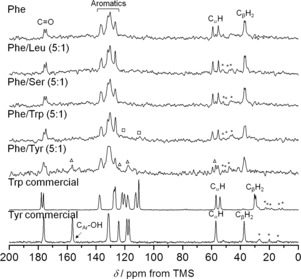
^1^H‐^13^C CP/MAS NMR spectra of hydrogels of Phe, Phe/Leu (5:1), Phe/Ser (5:1), Phe/Trp (5:1) and Phe/Tyr (5:1) acquired with MAS rates of 8.5 kHz, and reference solid powders of the commercially available Trp (CSD ref. QQQBTO03)^20^ and Tyr (CSD ref. LTYROS02)^20^ acquired with MAS rates of 10.5 kHz. Rectangles and triangles highlight the presence of rigid elements of Trp and Tyr, respectively. Asterisks represent spinning sidebands.

^1^H‐^13^C cross‐polarisation magic angle spinning (CP/MAS) solid‐state NMR experiments of hydrogels provided information on the rigid components, which are characterised by strong heteronuclear dipolar couplings that efficiently transfer magnetisation between ^1^H and ^13^C spins.^21^ Spectra acquired on both wet (Figure 7) and dried hydrogels were very similar (Figure S5 in Supporting Information). Not surprisingly, the spectra of dried hydrogels presented a significant improvement in signal‐to‐noise ratio due to the increased percentage content of the gelator during the measurements. The ^1^H‐^13^C CP/MAS NMR spectrum of the hydrogel of Phe displayed peak splitting for each carbon site, due to the presence of two magnetically inequivalent molecules in the asymmetric unit of the monohydrate phase of Phe (Figure 7).^17^ Upon introduction of additives, the same chemical shift values and splitting patterns were observed for all Phe peaks (Table S4 in Supporting Information), confirming that the Phe monohydrate phase was maintained as the supramolecular structure forming the hydrogel fibres, which is in excellent agreement with PXRD data.

Additional peaks detected for hydrogels of Phe/Trp and Phe/Tyr were assigned to Trp and Tyr carbons, respectively (Figure 7). Significant broadening of Trp and Tyr peaks was observed in the hydrogels as compared to the sharp peaks of the reference crystalline powders of Trp and Tyr; an indication of the participation of these molecules in less ordered structures. In dried hydrogels of Phe/Tyr, the Tyr peaks appeared as much sharper resonances with chemical shift values identical to the reference solid powder of Tyr (Figure S5 in Supporting Information). This is consistent with the PXRD patterns, indicating the existence of a fraction of crystalline Tyr in the gel matrix. Upon drying, this fraction is increased as Tyr precipitates out of solution.

^1^H‐^13^C CP/MAS NMR spectra of hydrogels of Phe/Leu and Phe/Ser did not exhibit any carbon peaks for Leu or Ser; an indication these remained in a highly mobile state. Overall, these findings strongly suggested Leu and Ser were not incorporated in the rigid elements of the fibrous networks.

### Investigation of semi‐solid components

High‐resolution magic angle spinning (HR‐MAS) NMR spectra can be used to probe semi‐solid components at the interfaces between the hydrogel fibres and the pools of water. According to Iqbal et al., the application of a diffusion filter in pulsed‐field gradient (PFG) experiments eliminates the contribution of fast moving molecules, hence enabling us to filter out free gelator molecules from ^1^H HR‐MAS NMR spectra.^22^ PFG HR‐MAS NMR experiments were conducted for single and multi‐component hydrogels. However, the relatively small dimensions of the molecules under study prevented differentiation between the rigid moieties incorporated in the gel fibres from the mobile regions exposed to water.

**Figure 8:**
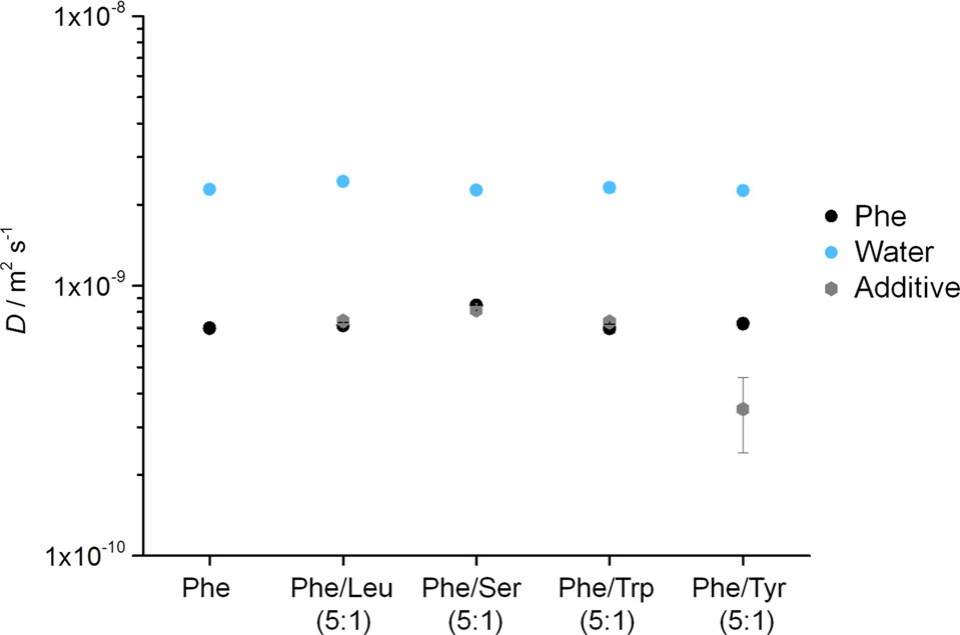
Apparent self‐diffusion coefficients (*D*) calculated from ^1^H PFG HR‐MAS NMR experiments for water, Phe, and additive molecule in the hydrogels of Phe, Phe/Leu (5:1), Phe/Ser (5:1), Phe/Trp (5:1) and Phe/Tyr (5:1), respectively, acquired with MAS rates of 1 kHz at 298 K. Tyr presented poor signal‐to‐noise, preventing accurate determination of its apparent self‐diffusion coefficient.

The dynamic behaviour of valine‐based organogels has been described by Escuder et al. The authors discussed various equilibria between free gelator molecules and oligomeric aggregates in solution, in the light of different time scales of several NMR experiments.^23^ In an attempt to distinguish different species, apparent self‐diffusion coefficients (*D*) were determined (Figure 8). These *D* values differ from those calculated from solution‐state experiments, since diffusion of molecules is affected by sample rotation.^24^ The curves (Figure S7 in Supporting Information) were best fitted to a mono‐exponential function, reflecting the presence of a single molecular diffusion regime (*D* values can be found in Table S5).

The values of apparent self‐diffusion coefficients for water and Phe were very similar for all hydrogels (Figure 8) and are consistent with those reported for supramolecular gels.^24^, ^25^ In hydrogels of Phe/Leu and Phe/Trp, the *D* values for Leu and Trp were in the same range as Phe (*D*≈7×10^−10^ m^2^ s^−1^). On the contrary, a higher apparent self‐diffusion coefficient was determined for Ser, in the hydrogels of Phe/Ser. This indicated that Ser exists mainly as a free mobile molecule in the pools of solvent. Tyr presented poor signal‐to‐noise (due to significant overlap with Phe aromatic peaks and poor water solubility of Tyr), preventing accurate determination of its apparent self‐diffusion coefficient.

### Dynamics of molecules in solution

**Figure 9:**
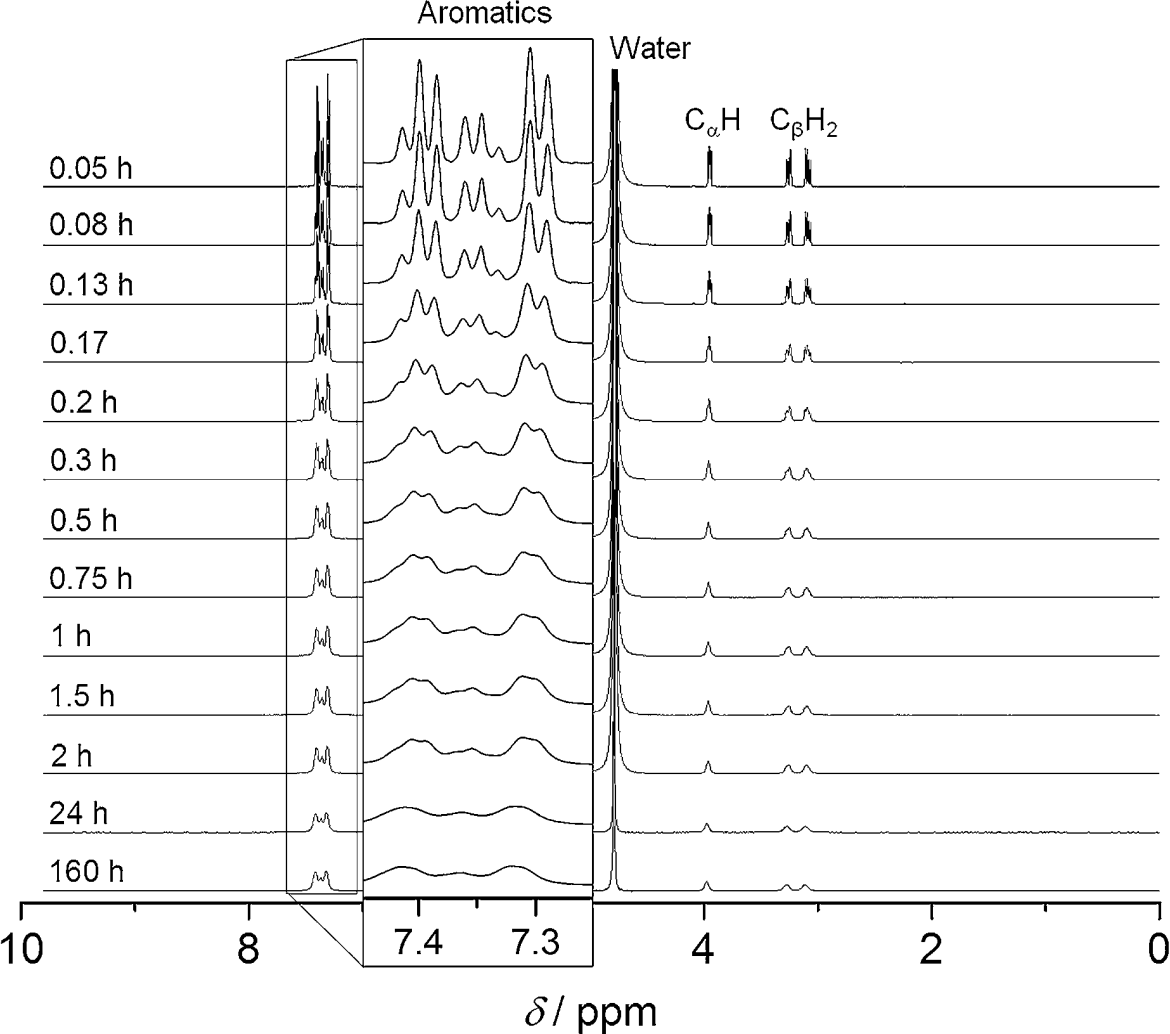
Kinetics of gelation of Phe monitored by the acquisition of ^1^H solution‐state NMR spectra over time, immediately after cooling down a hot solution of Phe (303 mm) (performed at 298 K).

Spectral variations, that is, reduced peak intensities, line broadening and chemical shift changes, can serve as an indication of the degree of incorporation of free molecules into the rigid components of gels, since these molecules become invisible to solution‐state NMR experiments.^23^ The kinetics of self‐assembly was monitored initially through the acquisition of several ^1^H solution‐state NMR spectra at room temperature immediately after cooling down a hot solution of Phe. Very sharp and intense peaks of Phe were recorded three minutes after quenching (Figure 9). Gradually, these peaks became broader and less intense, until a plateau was reached after 24 h, consistent with gelation.^23^ These changes in intensity of peaks indicated that ca. 40 % of Phe molecules formed the rigid hydrogel fibres (Table S6 in Supporting Information).

**Figure 10:**
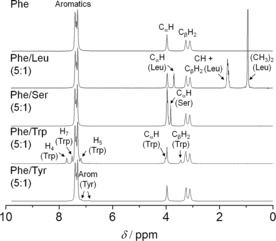
^1^H solution‐state NMR spectra of hydrogels of Phe, Phe/Leu (5:1), Phe/Ser (5:1), Phe/Trp (5:1) and Phe/Tyr (5:1) acquired 24 h after quenching hot solutions (303 mm). Tyr peaks displayed much lower intensities than expected according to the composition, as Tyr precipitates under the experimental conditions.

The consequences of introducing additive molecules were reflected even at early stages of self‐aggregation. The kinetics of gelation of Phe, that is, the evolution of peak intensity and line broadening with time, were modified to different extents in multi‐component hydrogels (Figure S8). 24 h ageing of these hydrogels led to reduced peak intensities for Trp and Tyr. However, no significant peak variations were identified for either Leu or Ser (Figure 10, Tables S5 and S6). Interestingly, intensities of Phe peaks increased upon the addition of Ser, in hydrogels of Phe/Ser (Table S6), indicating that the presence of Ser led to lower concentrations of Phe molecules in the fibres.

The intermolecular interactions responsible for formation of the hydrogel network were probed using nuclear Overhauser effect (nOe) spectroscopy (NOESY). Negative nOe enhancements were detected in ^1^H‐^1^H 2D NOESY NMR spectra for Phe protons in Phe hydrogels (Figure S9 in Supporting Information). This is characteristic of large molecules which transfer magnetisation efficiently through dipolar interactions.^26^ Since these Phe‐based hydrogel systems are composed exclusively of low molecular weight species, these findings indicated that molecules in solution contain information from the fibrous network due to their fast dynamics of exchange in the NMR frequency time scale. The phenomenon of solution‐state NMR spectra containing information from the hydrogel fibres due to fast molecular exchange between solution and gel states has been described previously^23^, ^26^ and further confirmed by the determination of a single diffusion regime (see above). Strong negative cross‐peaks were also recorded between Phe and Trp or Tyr, supporting the hypothesis that Trp and Tyr were in close proximity with Phe due to their incorporation in the Phe/Trp and Phe/Tyr hydrogel fibres. The presence of weak negative cross‐peaks between Phe and Leu indicated spatial proximity between both molecules. However, the absence of Leu peaks in CP/MAS NMR spectra suggested these were interactions occurring merely at the gel/solution interfaces. This is in good agreement with the lower apparent self‐diffusion coefficient determined for Leu in the hydrogel of Phe/Leu in comparison with solutions of Leu. Contrarily, no cross‐peaks were observed between Phe and Ser in the hydrogel of Phe/Ser.

**Figure 11:**
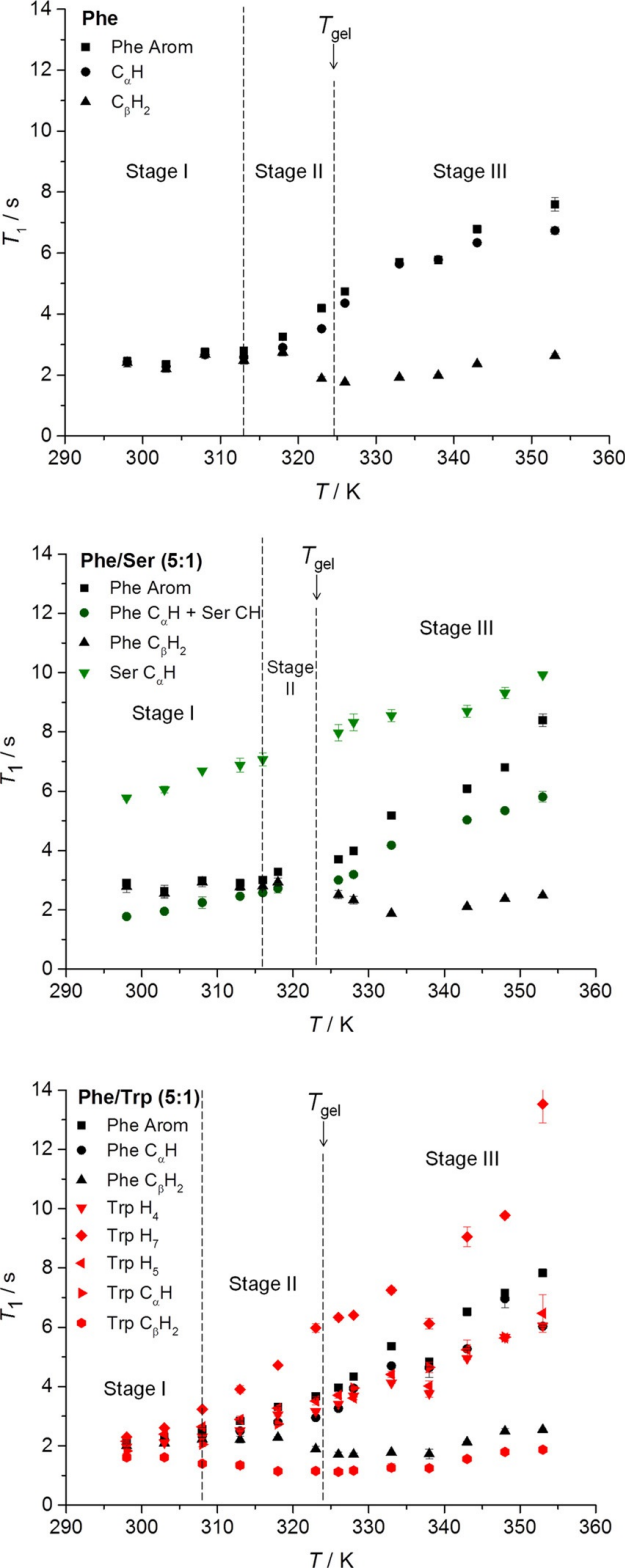
^1^H solution‐state NMR *T*_1_ times of Phe, Ser and Trp in hydrogels of Phe, Phe/Ser (5:1) and Phe/Trp (5:1), recorded from 298 to 353 K.

Further insight into gelation mechanisms and molecular motional states was obtained by ^1^H solution‐state NMR longitudinal relaxation times (*T*_1_) measured during the gel‐to‐solution transitions of single and multi‐component hydrogels (Figures 11 and S 10). VT NMR measurements of ^1^H *T*_1_ times indicate the systems are in the “fast tumbling regime”, also corroborated by the decreased line widths with increasing temperature.^27^ Three distinct stages in the transformation of the hydrogels were identified during variable temperature measurements of ^1^H *T*_1_ times. During stage I, the sample was in the gel state and ^1^H *T*_1_ times of Phe were similar for different ^1^H sites (*T*_1 Phe Arom_=2.45 s, *T*_1 Phe_ ${}_{C{}_{\alpha }H}$ =2.42 s and *T*_1_ ${}_{C{}_{\beta }H{}_{2}}$ =2.41 s, at 298 K), which correspond to averaged values resultant from exchange phenomena of the gelator molecules between solution and gel states. Bouguet‐Bonnet et al. reported a similar observation for single component organogels of a derivative of phenylalanine and naphthalimide.^26^ During stage II, a heterogeneous distribution of *T*_1_ times typical of Phe solutions was observed, reflecting the disassembly of the network and the presence of more Phe molecules in the solution state. The process of the molecular gel‐to‐solution transition, *T*_gel_^*^,^28^ usually occurs at temperatures below the macroscopically determined *T*_gel_ (here at 313 K). As temperature was raised further, gradual dissolution of the fibres led to progressive destruction of the hydrogel, which finally resulted in a loss of structural integrity (reflected by *T*_gel_). This temperature marked the beginning of stage III, after which the distribution of *T*_1_ times increased further and resulted in an overall increase of *T*_1_ times for Phe Arom and Phe C_α_H (*T*_1 Phe Arom_=7.59 s and *T*_1 Phe_ ${}_{C{}_{\alpha }H}$ =6.74 s, at 353 K). Sharpening of the peaks was also consistent with faster molecular motions occurring at higher temperatures. However, ^1^H *T*_1_ times for Phe C_β_H_2_ followed a different trend, with rapid decay throughout the range of temperatures and a minimum at 326 K (*T*_1_ ${}_{C{}_{\beta }H{}_{2}}$ =1.76 s). This minimum possibly reflected the point after which ^1^H *T*_1_ values corresponded mainly to fast tumbling molecules dissolved in solution.

For multi‐component hydrogels, ^1^H *T*_1_ times of Phe determined at 298 K increased according to the following trend: Phe/Trp < Phe/Tyr ≤ Phe ≤ Phe/Leu < Phe/Ser (Table S8 in Supporting Information). The strongest hydrogel, composed of Phe and Trp, presented the fastest relaxation rates for Phe Arom protons. The weakest material, formed by Phe and Ser, contains the highest concentrations of dissolved Phe, the slower relaxation profiles of which contribute to a higher averaged ^1^H *T*_1_ value. ^1^H longitudinal relaxation times may therefore mirror the strength of the hydrogel fibres, although such a comparison should only be performed when discussing systems with similar composition.

For all hydrogels, Phe ^1^H sites displayed an evolution of *T*_1_ times with temperature similar to the single component system. Furthermore, ^1^H *T*_1_ times for Trp and Tyr presented similar temperature dependence to those in hydrogels of Phe/Trp and Phe/Tyr, respectively. At low temperatures, ^1^H *T*_1_ times were similar for different ^1^H sites followed by a gradual dispersion of values as temperature was increased (Figures 11 and S10). The evolution of ^1^H *T*_1_ times for Trp and Tyr throughout the gelation processes proved that these additive molecules were incorporated in the network at lower temperatures and started tumbling faster, as free molecules, as temperature was raised. Hence, our *T*_1_ findings strongly support the claim that Trp and Tyr are intimately associated with Phe within the solid elements of the 3D network and in equilibrium with free molecules dissolved in the isotropic pools of water (i.e., bulk solvent).

In hydrogels of Phe/Leu and Phe/Ser, a linear evolution of ^1^H *T*_1_ times of Leu and Ser was observed throughout the range of temperatures with a distribution of values similar to solutions (Figures 11 and S10). In the gel state, Leu and Ser protons showed long *T*_1_ values and narrow peak line widths in ^1^H solution‐state NMR spectra (Figure 10), characteristic of fast molecular motions. These findings, in combination with the lack of peaks of Leu and Ser in ^1^H‐^13^C CP/MAS NMR spectra, are strong indications that these additive molecules remain essentially dissolved in pools of water surrounded by Phe fibres.

### Investigation of binding processes to the fibrous network by STD NMR

Exchange phenomena between molecules incorporated in the hydrogel network and those dissolved in the isotropic solution phase can be elucidated further using saturation transfer difference (STD) NMR experiments.

**Figure 12:**
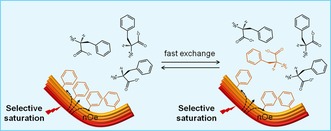
Proposed mechanism for transfer of saturation in STD NMR experiments performed with supramolecular hydrogels. Gelator molecules forming the network (bound state, left) are in fast exchange with those in the bulk solution phase (free state, right), allowing accumulation and detection of saturation in solution.

STD NMR spectroscopy is applied frequently to identify the functional groups of a ligand responsible for binding to its receptor (a protein, typically).^29^ This method relies on the transfer of saturation through cross‐relaxation from a large saturated protein to a small bound ligand.^30^ The potential of STD NMR in the studies of supramolecular gels has been recognised recently.^31^ For amino acid based hydrogels, we can consider the network as the supramolecular entity that can be saturated selectively. Such saturation might then be transferred through the nuclear Overhauser effect throughout the network and finally passed over intermolecularly to the bound gelator molecules. Dissociation of the weakly bound molecules from the network into the pools of water results in accumulation of saturation in the isotropic solution phase for molecules that exchange faster than their relaxation rates (Figure 12). This accumulation occurs due to the much longer longitudinal relaxation times for unbound fast tumbling molecules than for bound slow tumbling molecules.^32^ In this way, the STD difference spectrum will only exhibit signals of protons of gelator molecules that, being in solution, have been in contact with (and hence received saturation from) the fibrous network.

**Figure 13:**
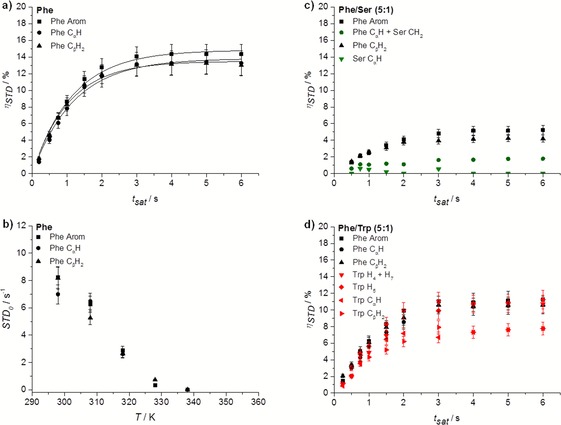
Build‐up curves of *η*_STD_ in hydrogels of a) Phe, c) Phe/Ser (5:1) and d) Phe/Trp (5:1) acquired at 298 K (STD_on_=0 ppm and STD_off_=40 ppm). The application of STD in the study of exchanging phenomena in supramolecular hydrogels was found to be reproducible with an error of 9.6 % (Supporting Information, Table S8). b) Initial slope values recorded from 298 to 338 K upon saturation of the network (STD_on_=0 ppm and STD_off_=40 ppm) in the hydrogel of Phe.

The observed mono‐exponential evolution of fractional STD response, *η*_STD_, with saturation time (build‐up curves) supported the presence of fast exchanging processes (on the NMR relaxation time scale) of Phe between free and bound states in the hydrogel of Phe (Figure 13 a). To analyse the experiments quantitatively, the initial slopes of the curves, *STD*_0_, were considered. This is also done to avoid potential effects of differences in longitudinal relaxation times and rebinding processes on the accumulation of saturation.^33^ No significant differences were observed between *STD*_0_ values of different proton sites of Phe, which prevented us from drawing conclusions about specific structural details on the bound state. Possibly the data are indicative of Phe molecules fully inserted in the fibres, in agreement with their relatively small dimensions.

STD NMR experiments were also conducted at variable temperature to monitor how exchange phenomena were affected throughout the gel‐to‐solution transitions. *STD*_0_ for Phe protons decreased rather linearly with increasing temperature (Figure 13 b). As the determined ^1^H *T*_1_ times were longer at higher temperatures, this reduction cannot be explained in terms of saturation losses by longitudinal relaxation. The significant reduction in *STD*_0_ observed was considered to reflect two processes: 1) the increase in the binding kinetics with temperature, reducing the fraction of bound species, and, more importantly, 2) the gradual dissolution of the supramolecular network, reaching full dissolution at 338 K. This temperature was higher than the macroscopically determined *T*_gel_, since the former reflects the dissolution of the supramolecular network (that acts as reservoir of magnetisation), whereas the latter describes the loss of structural integrity. Relaxation studies corroborated these findings, since 338 K was the temperature above which the distribution of ^1^H *T*_1_ values resembling that in solutions of Phe was observed (Figures 11 and S10). At very high temperatures, molecules form a pure solution with individual molecules behaving isotropically, and hence no STD signals were detected.

The ability of STD NMR to detect the exchange of gelator molecules between the network and the solution at temperatures above *T*_gel_ is particularly interesting. We therefore propose the application of variable temperature STD NMR experiments to supramolecular gels as a quick and robust tool to provide information at a molecular level on the different stages of dissolution of the 3D network. These results could pave the way for the use of STD NMR to understand non‐covalent supramolecular assembly processes that occur through nucleation (e.g., aggregation, gelation or crystallisation) and current investigations are underway in our research group.

The effect of introducing an additive was assessed through the evaluation of *η*_STD_ (Figures 13 and S 11) and *STD*_0_ (Table S9 in Supporting Information) in multi‐component systems. *η*_STD_ values of Phe protons decreased in all multi‐component hydrogels in comparison with the hydrogel of Phe. In contrast to classical exchange processes in protein‐ligand interactions (in which there is a determined number of binding sites decorating the surface of the protein), in a supramolecular system such a decrease in saturation transfer may be attributed to several phenomena, which are discussed below.

Lower values of *η*_STD_ might result from: 1) decreased binding strength of Phe, due to perturbations of the network structure from the binding of the additive, or 2) from competition of the additive with Phe for the interaction sites, assuming the network interaction sites remain the same in the presence and in the absence of the additive. In both cases, there will be an increased amount of Phe in solution, which reduces the fraction of bound molecules, and hence the STD (Equation S1 in Supporting Information). On the other hand, slow exchange on the NMR relaxation time scale, associated with strong binding, would result in longer residence time in the network‐bound state, lower accumulation of saturation in solution and, therefore, lower STD signals.

In the hydrogels of Phe/Trp and Phe/Tyr, Trp and Tyr, aromatic protons displayed *η*_STD_ values in the same range as protons of Phe (Figures 13 d and S11 f). These experiments provided evidence supporting the claim that Trp and Tyr are in fast exchange between the free and network‐bound states. More importantly, the similarity between *η*_STD_ values for Phe and Trp or Tyr can be interpreted in terms of the strength of binding of these additives to the network. Under these conditions, if Trp and Tyr had similar affinities for the network as Phe, their *η*_STD_ values would be much larger than the *η*_STD_ values of Phe. Their lower *η*_STD_ values, along with the reduction of *η*_STD_ values of Phe, suggested that Trp and Tyr were stronger binders to the network than Phe, accumulating lower saturation in solution. In this way, the combination of CP/MAS NMR findings, ^1^H *T*_1_ measurements, nOe‐based experiments and solution‐state NMR spectral variations allowed us to conclude confidently that Trp and Tyr make part of the rigid components of the fibrous network.

The lower *η*_STD_ observed for Phe in hydrogels of Phe/Trp and Phe/Tyr could be interpreted incorrectly as an indication of an increase in the amount of Phe in the free state, resulting from the competitive binding between Phe and the additive. This is not the case for two reasons. Firstly, supramolecular gels do not have a well‐defined number of interaction sites, unlike binding pockets in proteins. The hydrogel network is a dynamic assembly, and binding of additives will not just passively compete with Phe, but can alter the structure and dynamics of the interaction sites. Secondly, this interpretation is contradictory to the presence of lower concentrations of Phe in solution (Table S6 in Supporting Information) and the enhanced resistance to deformation found for the hydrogel of Phe/Trp (Figure 5). Strengthening of supramolecular gel fibres is usually associated with an increased number of gel forming molecules, leading to formation of more fibres and interfibrillar connections. In light of these findings, the lower *η*_STD_ for Phe occurs due to modification of the strength of the interactions of Phe with Trp or Tyr rich domains located in the gel fibres, resulting in different rates of exchange.

The low STD response recorded for Leu in the hydrogel of Phe/Leu confirmed a weak interaction of Leu with the gel/solution interfaces, in agreement with the findings from NOESY experiments. Additionally, there was no evidence in CP/MAS NMR experiments of the incorporation of Leu in the rigid components of the network. In the case of Ser‐based hydrogels, the absence of *η*_STD_ for Ser indicated absence of binding to the network, in agreement with ^1^H solution‐state NMR experiments in which Ser protons appeared as sharp intense resonances with long *T*_1_ times, suggestive of fast molecular tumbling. The increased amount of dissolved Phe observed in the ^1^H spectra of the hydrogel of Phe/Ser explains the decrease in *η*_STD_ values for Phe protons.

Hence, the NMR experimental data on Phe/Ser hydrogels indicate that Ser has disruptive effects on the Phe network, explaining the markedly lower *G′* value measured. Although Phe/Ser interactions have previously been described,^34^ NOESY and STD NMR experiments showed that Ser does not interact with the Phe network in the gel state. In addition, we observed slower kinetics of gelation for the hydrogel of Phe/Ser hydrogel, monitored by the evolution of Phe peaks with time (Figure S8). Altogether, the data strongly support that Phe/Ser interactions are likely to be predominant during the nucleation phase of the gelation processes.

Our study emphasises the dynamic complexity of multi‐component hydrogels: the effect of additive molecules on hydrogels might not result only from their interactions with the final gel product, but also from modifications introduced during nucleation and/or growth processes. Therefore, when assessing the impact of additive molecules on hydrogels by means of STD NMR studies, observations from CP/MAS experiments should also be taken into account. If the additive molecule shows a build‐up of saturation and peaks in CP/MAS spectra of hydrogels, they might be incorporated into the rigid fibres. If the additive molecule accumulates saturation in solution but no peaks are observed in CP/MAS experiments, there is no incorporation but weak interactions occurring only at the surface of the fibres.

In conclusion, STD NMR experiments confirmed that all investigated supramolecular hydrogels were dominated by fast exchange processes between free and bound gelator molecules, characteristic of a highly dynamic environment. The combination of STD NMR and CP/MAS NMR experiments is a very powerful approach to monitor the incorporation of additives into the hydrogel supramolecular fibrous network. These studies have proven capable of providing insight into the dynamics of soft materials, even when the solid component is crystalline.

## Conclusions

Solid and solution‐state NMR experiments have provided mechanistic insights into the processes of supramolecular multi‐component gel formation and the dynamics of the resultant materials. The development of multi‐component systems proved to be an elegant strategy to modify the rheological properties of the “gel‐crystal” supramolecular system. It was possible to correlate the strength of the gel with the composition of the gel fibres and solid/liquid interface in the presence of additive gelator molecules. Modification of the kinetics of gelation, due to interference with nucleation and fibre growth, changed the concentration of gelator molecules incorporated within the rigid structures. This indicates differences in the gelation mechanism of multi‐component systems, which in turn altered the materials resistance to deformation. The addition of Ser resulted in weaker materials, due to an effectively lower concentration of Phe in the solid fibres. In contrast, Trp was proven to contribute to stabilisation of the 3D network, as a higher content of molecules incorporated in the solid structures led to a higher number of fibres and interfibrillar cross‐links, resulting in a material more resistant to deformation.

These studies confirmed that only the combination of complementary analytical techniques, capable of probing several levels of self‐organisation and mobility regimes, can fully characterise the multiphasic character of supramolecular gels. Using CP/MAS NMR experiments and PXRD techniques, we were able to probe the rigid, structural elements of the fibrous network of supramolecular systems, both in wet and dried states, while solution‐state NMR experiments enabled us to monitor pre and post‐gelation aggregates of amino acids. More importantly, we demonstrated and discussed the potential of saturation transfer difference (STD) NMR experiments in understanding exchange dynamics and binding processes in multi‐component supramolecular hydrogels. The application of the presented approach enables better understanding of the role of guest molecules, such as drugs, co‐gelators or non‐gelating additives, in the self‐assembly processes of supramolecular soft materials, which is of paramount importance in knowledge based design of new functional materials.

## Experimental Section

### Materials

Reagent grade (>98 %) l‐leucine, l‐phenylalanine, l‐serine, l‐tryptophan, l‐tyrosine, and hexamethylbenzene (HMB) were purchased from Sigma–Aldrich. Deuterium oxide, 4,4‐dimethyl‐4‐silapentane‐1‐sulfonic acid (DSS), and tetramethylsilane (TMS) were purchased from Goss Scientific. Milli‐Q water was obtained with a Thermo Scientific Barnstead NANOpure purification system coupled to a Barnstead hollow fibre filter.

### Preparation of hydrogels

Hydrogels were prepared by dispersing amino acids in 1 mL of light (H_2_O) or heavy (D_2_O) water in a glass vial (2 cm diameter) promoting dissolution with a vortex mixer for 30 s. These samples were heated up to 363 K with a hot plate and immediately quenched in a water bath at 273 K. The hydrogel samples were left resting overnight at room temperature and analysed 24 hours after preparation. Hydrogelation was assessed through the vial inversion test. Temperature of gelation was determined by heating up the hydrogel samples with a hot plate at a heating rate of 1 K min^−1^ while performing vial inversion tests. The resulting hydrogels were dried under vacuum.

### Scanning electron microscopy

Morphology of the hydrogel fibres was determined using scanning electron microscopy (SEM). SEM experiments were carried out using a Jeol JSM‐5900 LV Oxford instrument with an accelerating voltage of 2.1 kV. Hydrogels were mounted on aluminium stubs with double sided carbon adhesive and allowed to dry before analysis. Dried samples were then gold‐coated using a Quorum Technologies Polaron SC7640 gold sputter coater.

### Atomic force microscopy

Nanoscale morphology of the hydrogel fibres was determined using atomic force microscopy (AFM). AFM experiments were conducted using a JPK NanoWizard instrument with a silicon cantilever at a nominal spring constant of 40 N m^−1^, operated with intermittent contact for imaging. Hot solutions (ca. 10 μL) were pipetted onto a sample holder and allowed to dry before analysis.

### Rheology

Resistance of fibres to mechanical stress was investigated using rheology. Measurements were performed using a Bohlin Gemini HR^nano^ Rotonetic 2 drive, equipped with a Julabo F12 water cooler and circulator controlling the temperature of the bottom Peltier plate, and an aluminium cone and plate geometry system (truncated 4/40 cone, 4° cone angle and 40 mm diameter). Hot solutions (ca. 1.5 mL) were pipetted into a 300 μm gap, with the temperature of the plate maintained at 323 K for sample preparation. The temperature was then lowered to 293 K, covered with a solvent trap to prevent solvent evaporation, and the hydrogels were left stabilizing for 1 h. Phase angle, storage and loss moduli were monitored and recorded as a function of frequency and stress. All samples were subjected to frequency sweeps in the range of 0.1 to 100 Hz and applied stress of 700 Pa, as well as stress amplitude sweeps in the range of 500 to 10 000 Pa.

### Powder X‐ray diffraction

Long‐range ordering of the obtained hydrogels was investigated using powder X‐ray diffraction (PXRD) with a Thermo Scientific ARL XTRA powder diffractometer under Cu_Kα_ radiation (*λ*=1.54 Å). Samples were analysed in the 2*θ* range of 5 to 36°, with a step size of 0.01° and a scan time of 6 s. Hydrogels (ca. 1 mL) and dried samples were placed onto stainless steel sample holders and analysed immediately to prevent dehydration.

### Nuclear magnetic resonance spectroscopy

#### Solid‐state NMR spectroscopy

Structure and dynamics of hydrogels were characterised using nuclear magnetic resonance spectroscopy. Solid‐state NMR experiments were performed using a Bruker Avance III spectrometer equipped with a 4 mm triple resonance probe operating at frequencies of 400.23 MHz (^1^H) and 100.64 MHz (^13^C). Hydrogels were prepared by pipetting 40 μL of hot solutions into Kel‐F plastic inserts and allowing them to cool down and gelate inside the insert. ^1^H‐^13^C cross‐polarisation magic angle spinning (CP/MAS) NMR experiments of reference solid powders were acquired using 128 scans at an MAS rate of 10.5 kHz, a recycle delay of 30 s and contact time of 2 ms. ^1^H‐^13^C CP/MAS NMR spectra of hydrogels were acquired using 8192 scans and an MAS rate of 8.5 kHz with a recycle delay of 20 s and contact time of 2 ms. The Hartmann–Hahn matching condition was established with hexamethylbenzene (HMB). All spectra were referenced using tetramethylsilane (TMS) as a standard.

#### HR‐MAS NMR spectroscopy

High‐resolution magic angle spinning (HR‐MAS) NMR experiments were performed using a Bruker Avance III spectrometer operating at a ^1^H frequency of 400.23 MHz equipped with a 4 mm double resonance probe and a *z*‐gradient coil reaching a maximum field gradient intensity of 49.5 G cm^−1^. Hydrogels were prepared by pipetting 40 μL of hot solutions into Kel‐F plastic inserts and allowing them to cool down and gelate inside the insert. All experiments were carried out with hydrogels prepared in D_2_O. Pulsed‐field gradient (PFG) NMR experiments were carried out using the stimulated echo and longitudinal eddy current delay diffusion‐filtered pulse sequence (ledgpgp2s), with 5 to 95 % of the maximum gradient intensity. A smoothed‐square shaped gradient (SMSQ10.100) was used. A diffusion delay (Δ) of 70 ms, a diffusion gradient length (δ) of 1 ms, a recycle delay of 2 s and MAS rates of 1 kHz were used in each experiment. Attenuated peak intensities were plotted against the *b* factor, *b*=−*γ* ^2^ *g* ^2^ *δ* ^2^(Δ−$\frac{\delta }{3}$ ).^21^ Apparent self‐diffusion coefficients (*D*) were obtained from the mathematical fitting of the resulting curves to the mono‐exponential function [Eq. (1)]:

(1) $$I=I_{0}e^{-D\gamma^{2}g^{2}\delta^{2}\left(\Delta -\frac{\delta }{3}\right)}$$

where *I* is the observed intensity, *I*_0_ is the unattenuated signal intensity and γ is the gyromagnetic ratio of the observed nucleus.^21^

#### Solution‐state NMR spectroscopy

Solution‐state NMR experiments were performed using a Bruker Avance I spectrometer operating at a ^1^H frequency of 499.69 MHz equipped with a 5 mm probe. Hydrogels were prepared by pipetting 600 μL hot solutions into NMR tubes and allowing them to cool down and gelate inside the tube. All experiments were carried out with hydrogels prepared in D_2_O. Variable temperature (VT) experiments were carried out in the range of temperature from 298 to 353 K, allowing thermal stabilisation of the sample for 15 min prior to spectra acquisition. ^1^H NMR spectra were acquired using excitation sculpting for water suppression (zgespg) with a recycle delay of 10 s. DSS was used as an internal standard inside a coaxial insert.

^1^H longitudinal relaxation times (*T*_1_) were measured using a standard inversion recovery pulse sequence with a recycle delay of 10 s. 16 points were recorded at variable time delays ranging from 0.1 to 20 s. The evolution of intensities was fitted mathematically to the mono‐exponential function [Eq. (2)]:

(2) $$M_{z}\left(\tau \right)=M_{0}*\left[1-e^{\left(\frac{-\tau }{T_{1}}\right)}\right]$$

where *M*_z_ is the *z*‐component of magnetisation, *M*_0_ is the equilibrium magnetisation and *τ* is the time delay.^35^

Saturation transfer difference (STD) NMR experiments were performed with selective saturation of a given ^1^H frequency by a train of 40 Gaussian pulses with the duration of 50 ms each (stddiffgp19.2), acquired with a constant total experiment duration of 6 s. STD spectra were created by the subtraction of an on‐resonance spectrum (STD_on_), in which a spectral region was selectively saturated, from an off‐resonance spectrum (STD_off_), acquired with no selective saturation. STD_on_ spectra were acquired at a saturation frequency of 0 ppm (where only resonances of the network can be encountered), whereas the STD_off_ saturation frequency was set for 40 ppm. Interleaved acquisition of STD_on_ and STD_off_ spectra was performed as a pseudo‐2D experiment to minimise artefacts caused by variations throughout the experiment. Each pair of experiments was acquired at variable saturation times ranging from 0.25 to 6 s. Signal intensity in the difference spectrum relative to the signal intensity in the STD_off_ spectrum was used to determine the fractional STD response, *η*_STD_ [Eq. (3)]:

(3) $$\eta_{STD}=\frac{I_{0}-I_{SAT}}{I_{0}}\times 100=\frac{I_{STD}}{I_{0}}\times 100$$

where *I*_0_ is the signal intensity from the STD_off_ spectrum, *I*_SAT_ is the signal intensity from the STD_on_ spectrum and *I*_STD_ is the signal intensity from the difference spectrum.^36^ STD build‐up curves were fitted mathematically to the mono‐exponential function STD_(tsat)_=STD^max^(1−*e* ${}^{\left(-\kappa {}_{\mathrm{sat}}\cdot t{}_{\mathrm{sat}}\right)}$ ), where STD^max^ is the maximum possible STD at very long saturation times, *κ*_sat_ is the saturation rate constant and t_sat_ is the saturation time. Initial slope values of the build‐up curves, STD_*0*_, were obtained from the product STD^max^×*κ*_sat_.^33^

## Conflict of interest

The authors declare no conflict of interest.

## Supporting information

As a service to our authors and readers, this journal provides supporting information supplied by the authors. Such materials are peer reviewed and may be re‐organized for online delivery, but are not copy‐edited or typeset. Technical support issues arising from supporting information (other than missing files) should be addressed to the authors.

Supplementary
