# Supplementary material for: Supramolecular Amino Acid Based Hydrogels: Probing the Contribution of Additive Molecules using NMR Spectroscopy
Source: Chemistry. 2017 May 23;23(33):8014–24. doi: 10.1002/chem.201700793 (PMC5575562; doi:10.1002/chem.201700793)
Supplement: Supplementary file 1 — Supplementary [file CHEM-23-8014-s001.pdf]

CHEMISTRY
A European Journal

Supporting Information

Supramolecular Amino Acid Based Hydrogels: Probing the Contribution of Additive Molecules using NMR Spectroscopy

Susana M. Ramalhete,[a] Karol P. Nartowski,[a, c] Nichola Sarathchandra,[a] Jamie S. Foster,[b] Andrew N. Round,[a] Jesús Angulo,[a] Gareth O. Lloyd,*[b] and Yaroslav Z. Khimyak*[a]

chem_201700793_sm_miscellaneous_information.pdf

SUPPLEMENTARY INFORMATION

Table of contents

1. pH of suspensions 3
2. Temperature of gelation 4
3. Thermogravimetric analysis 5
4. Atomic force microscopy 6
5. Rheology 7
6. Microscopy 8
7. Powder X-ray diffraction 9
8. NMR spectroscopy 10
   8.1. Solid-state NMR spectroscopy 10
   8.2. HR-MAS NMR spectroscopy 12
   8.3. Solution-state NMR spectroscopy 14
      8.3.1. ^{1}H-^{1}H 2D NOESY experiments 16
      8.3.2. Longitudinal relaxation times experiments 18
      8.3.3. STD NMR experiments 19

1. pH of suspensions

pH values were monitored upon the addition of other amino acids to suspensions of Phe in water, due to its importance in dictating non-covalent interactions and, therefore, self-assembly processes. No significant differences were found between the samples under study.

Table S1. pH values of suspensions of Phe, Phe/Leu (5:1), Phe/Ser (5:1), Phe/Trp (5:1) and Phe/Tyr (5:1) and dissociation constants for the molecules under study.

Suspension	pH	p*Ka*[1]	p*Kb*[1]	p*Kc*[1]
**Phe**	6.45	Phe: 2.18	Phe: 9.09	-
**Phe/Leu (5:1)**	6.52	Leu: 2.32	Leu: 9.58	-
**Phe/Ser (5:1)**	6.44	Ser: 2.13	Ser: 9.05	-
**Phe/Trp (5:1)**	6.49	Trp: 2.38	Trp: 9.34	-
**Phe/Tyr (5:1)**	6.27	Tyr: 2.24	Tyr: 9.04	Tyr: 10.10

2. Temperature of gelation

The temperature above which hydrogels were broken (i.e. loss of structural integrity reflected by ability to flow under inversion) was defined as the gel-to-solution transition (T_{gel}). Temperature of gelation was determined by heating up the hydrogel samples with a hot plate at a heating rate of 1 K min^{-1}. Hydrogelation was then assessed through the vial inversion test.

Table S2. Gel-to-solution transition temperatures (T_{gel}) of hydrogels of Phe, Phe/Leu (5:1), Phe/Ser (5:1), Phe/Trp (5:1) and Phe/Tyr (5:1).

Hydrogel	T_{gel} / K
**Phe**	323.6 – 326.6
**Phe/Leu (5:1)**	323.5 – 324.5
**Phe/Ser (5:1)**	323.5 – 324.1
**Phe/Trp (5:1)**	322.2 – 326.9
**Phe/Tyr (5:1)**	324.8 – 325.0

3. Thermogravimetric analysis

Thermogravimetric analysis (TGA) was carried out using a TGA Q5000 TA instrument by placing *ca.* 2 mg of dried samples onto platinum pans. Samples were heated from 298 to 423 K with a heating rate of 2 K min^{-1}.

The weight loss observed in thermogravimetric analysis of dried hydrogels was assigned to evaporation of physisorbed water retained after the drying process.

Table S3. Weight loss of dried hydrogels upon heating up to 423 K, using a heating rate of 2 K min^{-1}.

	Weight Loss / %
**Phe**	2.4
**Phe/Leu (5:1)**	2.5
**Phe/Ser (5:1)**	2.5
**Phe/Trp (5:1)**	1.8
**Phe/Tyr (5:1)**	1.8

4. Atomic force microscopy

Widths of fibrillar features were measured directly from AFM images in intermittent contact mode. n = 83 measurements, taken from 6 different images (5 x 5 μm, 512 pixels per line). A normal distribution fitted to this histogram had a peak value of 437 ± 16 nm.

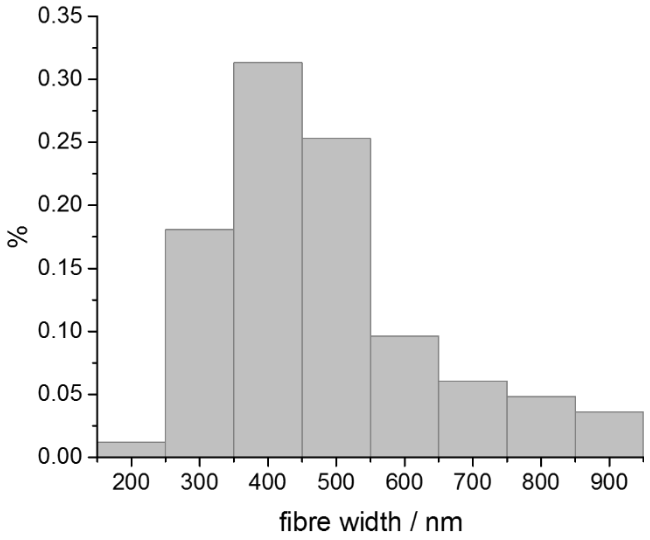

Figure S1. Histogram of measured widths in AFM experiments for the hydrogel of Phe, with an average width of 437 ± 16 nm.

5. Rheology

Phase angle, storage and loss moduli were monitored and recorded as a function of stress. All samples were subjected to stress amplitude sweeps in the range of 500 to 10000 Pa. An oscillatory torque was imposed with a fixed frequency over a range of shear stress amplitudes.

The hydrogel showed a typical *G′* value, essentially constant below the critical value of oscillatory torque (‘‘yield stress’’). At this yield stress point, the sample starts to flow or there is slippage between the interface of the rheometer and the hydrogel. No trends or conclusions could be drawn from this data due to this latter fact. Hydrogel materials often exude water (syneresis) resulting in uncontrollable slippage. Attempts were made to act against this but inconsistent data with little or no trends was obtained. The data below show general results with gels showing little differences, within error of normal data collection on the rheometer utilised for experiments.

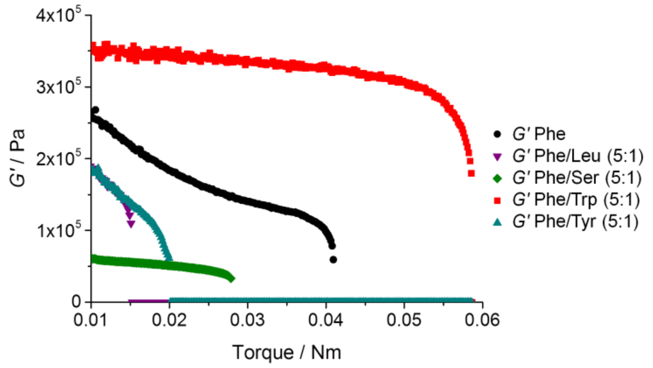

Figure S2. Storage modulus (*G*') at increasing stress sweeps for the hydrogels of Phe, Phe/Leu (5:1), Phe/Ser (5:1), Phe/Trp (5:1) and Phe/Tyr (5:1).

6. Microscopy

Polarised light microscopy was carried out using a Leica DMLS2 with ×20 magnification coupled to a JVC colour video camera. 20 µL of hot solutions were pipetted onto a glass slide and allowed to gelate *in situ*.
The photomicrographs of the hydrogel of Phe/Tyr showed the presence of crystalline needles, which were attributed to insoluble crystals of Tyr. This phase was also detected using PXRD and ^{1}H-^{13}C CP/MAS NMR.

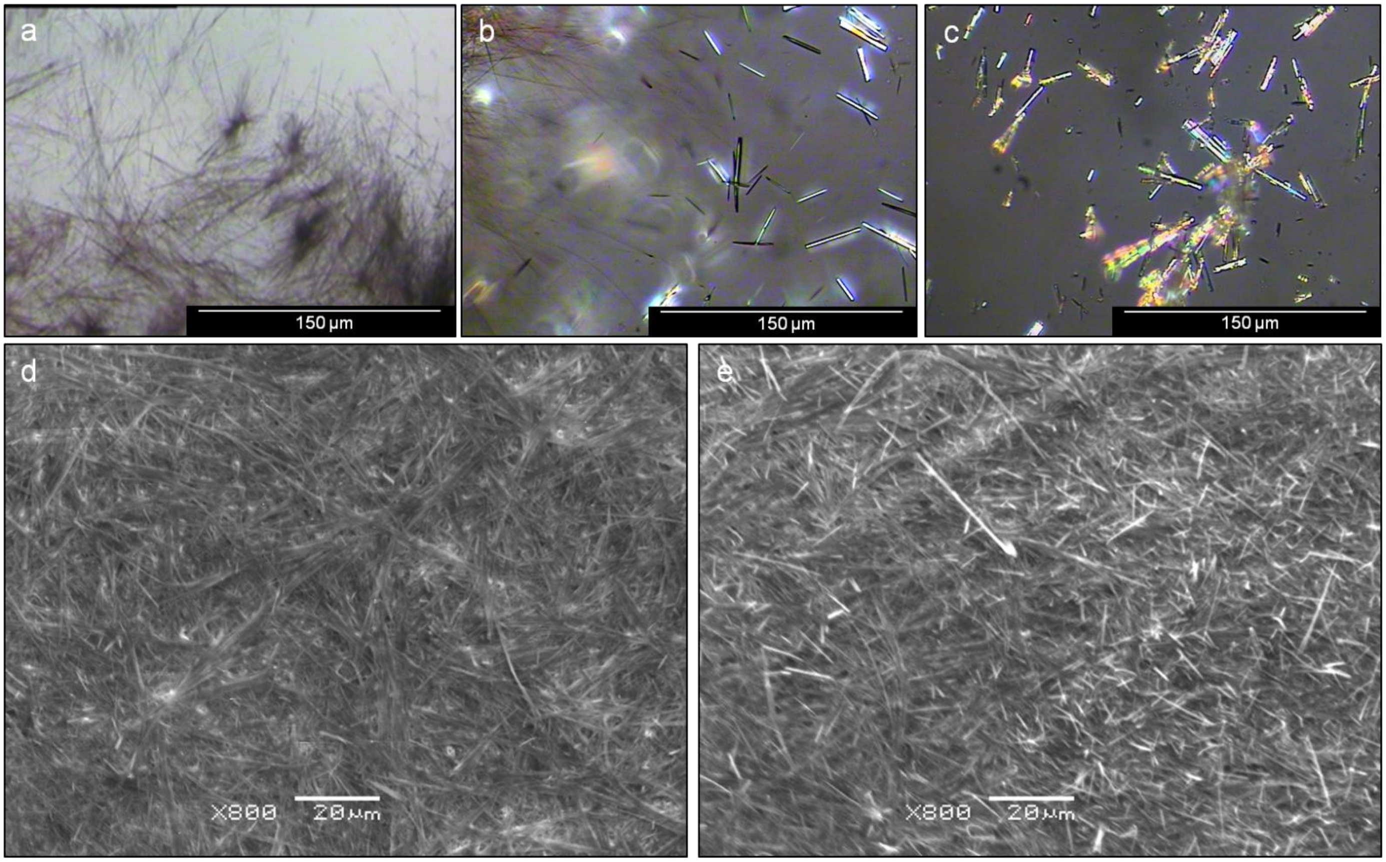

Figure S3. Polarised light microscopy images of the hydrogels of **a)** Phe and **b)** Phe/Tyr (5:1) (303 mM), and **c)** polarised light microscopy images of the commercially available Tyr (CSD ref. LTYROS02)[1]. SEM images of the dried hydrogels of **d)** Phe and **e)** Phe/Tyr (5:1) (303 mM). Insoluble white needle-like crystals of Tyr can be seen immersed in a network of entangled thin fibres.

7. Powder X-ray diffraction

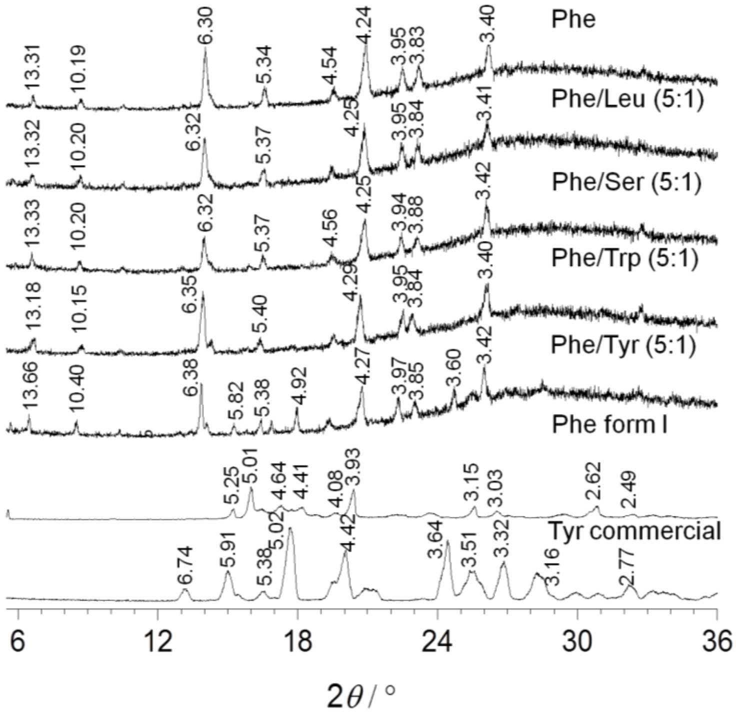

Figure S4. PXRD patterns of the hydrogels of Phe, Phe/Leu (5:1), Phe/Ser (5:1), Phe/Trp (5:1) and Phe/Tyr (5:1) and reference solid powders of the anhydrous form I of Phe and the commercially available Tyr (CSD ref. LTYROS02)[1].

8. NMR spectroscopy

8.1. Solid-state NMR spectroscopy

Hydrogels were prepared by pipetting 40 µL of hot solutions into Kel-F plastic inserts and allowed to cool down and gelate inside the insert. Dried hydrogels were packed inside zirconia rotors. ^{1}H-^{13}C CP/MAS NMR spectra of hydrogels were acquired using 8192 scans and an MAS rate of 8.5 kHz with a recycle delay of 20 s and contact time of 2 ms. ^{1}H-^{13}C CP/MAS NMR spectra of dried hydrogels were acquired using 2048 scans and an MAS rate of 10 kHz with a recycle delay of 20 s and contact time of 2 ms.

^{1}H-^{13}C CP/MAS NMR spectra were acquired on both wet and dried hydrogels, to compare the consequences of drying in the native tridimensional organisation of the hydrogels. Increased signal-to-noise was observed for dried materials. Interestingly, similar chemical shift values and peak splitting patterns were recorded in both physical states, indicating that experiments conducted with dried samples are able to reproduce the original structure of the hydrogel fibres in study.

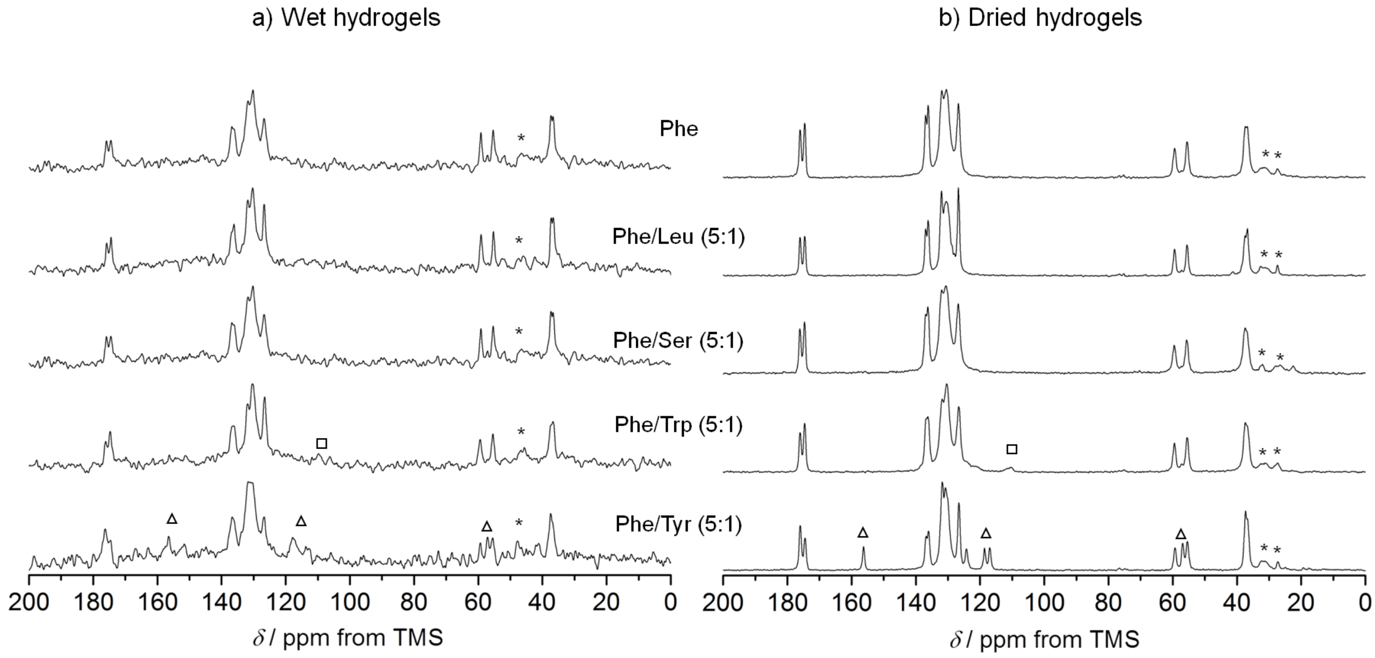

Figure S5. ^{1}H-^{13}C CP/MAS NMR spectra of **a)** wet and **b)** dried hydrogels of Phe, Phe/Leu (5:1), Phe/Ser (5:1), Phe/Trp (5:1) and Phe/Tyr (5:1) acquired with MAS rates of 8.5 and 10 kHz, respectively. Rectangles and triangles highlight the presence of rigid elements of Trp and Tyr, respectively. Asterisks represent spinning sidebands.

Table S4. ^{13}C chemical shifts from ^{1}H-^{13}C CP/MAS NMR spectra of hydrogels of Phe, Phe/Leu (5:1), Phe/Ser (5:1), Phe/Trp (5:1) and Phe/Tyr (5:1) acquired with MAS rates of 8.5 kHz. Carbon labelling scheme is shown for clarity.

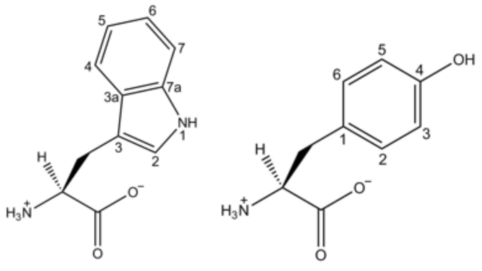

	δ / ppm from TMS				
	Phe	Phe/Leu (5:1)	Phe/Ser (5:1)	Phe/Trp (5:1)	Phe/Tyr (5:1)
**C=O**	176.0	176.0	176.0	176.0	176.0
	174.5	174.6	174.5	174.6	174.5
**Tyr C4-OH**	-	-	-	-	156.3
**Phe Aromatics**	136.9	136.9	136.8	136.7	136.8
	136.1	136.1	136.1	136.2	136.1
	132.0	131.8	131.8	131.7	131.8
	130.5	130.5	130.5	130.3	130.8
	126.7	126.7	126.7	126.6	126.6
**Tyr C3,5**	-	-	-	-	124.3
**Trp Aromatics**	-	-	-	120.8	-
**Tyr C2,6**	-	-	-	-	118.6
**Tyr C1**	-	-	-	-	117.0
**Trp Aromatics**	-	-	-	110.5	-
**Phe $C_{\alpha}H$**	59.4	59.4	59.4	59.4	59.3
	55.5	55.5	55.5	55.5	57.0
**Phe $C_{\beta}H_2$**	37.4	37.3	37.3	37.4	55.5
	36.7	36.8	36.8	36.7	37.4

8.2. HR-MAS NMR spectroscopy

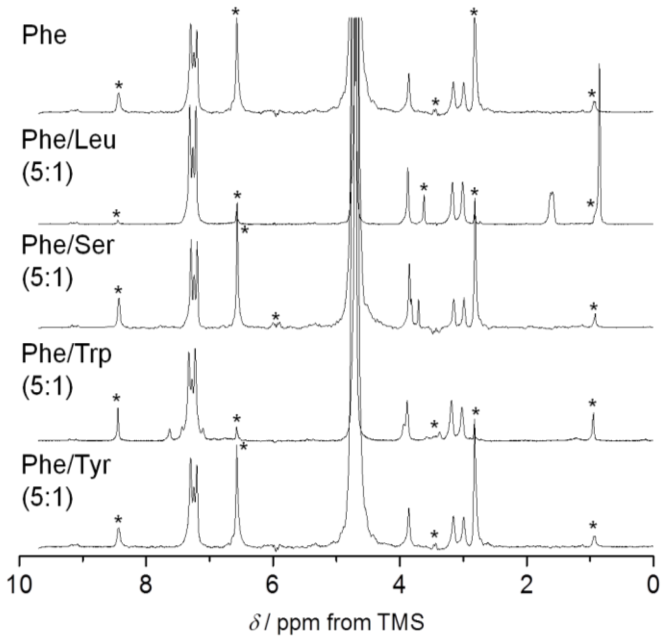

Figure S6. ^{1}H HR-MAS NMR spectra of hydrogels of Phe, Phe/Leu (5:1), Phe/Ser (5:1), Phe/Trp (5:1) and Phe/Tyr (5:1), acquired with MAS rates of 1 kHz at 298 K. Asterisks represent spinning sidebands.

Apparent self-diffusion coefficients (*D*) were determined from the mono-exponential fit of the evolution of peak intensity with increasing gradient strength.

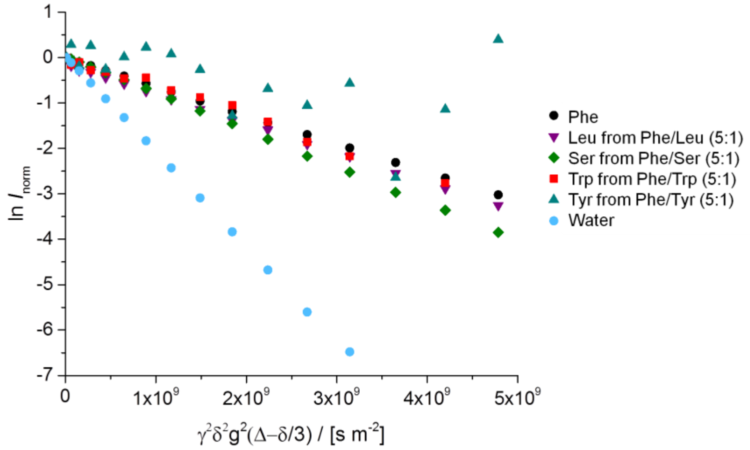

Figure S7. Evolution of ^{1}H PFG HR-MAS NMR normalised peak intensity with increasing gradient strength for water, Leu, Phe, Ser, Trp and Tyr in the hydrogels of Phe, Phe/Leu (5:1), Phe/Ser (5:1), Phe/Trp (5:1) and Phe/Tyr (5:1), acquired with MAS rates of 1 kHz at 298 K. Tyr presented poor signal-to-noise, preventing accurate determination of peak intensity.

Table S5. Apparent self-diffusion coefficients (*D*) calculated from pulsed-field gradient HR-MAS NMR experiments of hydrogels of Phe, Phe/Leu (5:1), Phe/Ser (5:1), Phe/Trp (5:1) and Phe/Tyr (5:1) acquired with a 5 to 95 % of the maximum gradient intensity (G_{max} = of 49.5 G cm^{-1}), a diffusion delay of 70 ms, a diffusion gradient length of 1 ms, a recycle delay of 2 s and MAS rates of 1 kHz at 298 K.

Hydrogel	Phe		Water		Additive molecule	
	$D \times 10^{10}$ / m^2 s^{-1}	$\delta \times 10^{10}$	$D \times 10^{9}$ / m^2 s^{-1}	$\delta \times 10^{9}$	$D \times 10^{10}$ / m^2 s^{-1}	$\delta \times 10^{10}$
**Phe**	6.98	0.13	2.28	0.01	-	-
**Phe/Leu (5:1)**	7.14	0.17	2.44	0.06	7.42	0.03
**Phe/Ser (5:1)**	8.47	0.03	2.27	0.01	8.12	0.11
**Phe/Trp (5:1)**	6.96	0.24	2.31	0.09	7.35	0.23
**Phe/Tyr (5:1)**	7.24	0.13	2.26	0.05	3.50 [a]	1.09

[a] Overlapped with Phe, preventing accurate determination

8.3. Solution-state NMR spectroscopy

Peak intensities from ^{1}H-NMR spectra can be correlated with concentration of diluted species when long enough recycle delays are applied. This can be used to derive the ratio between gelator molecules in the isotropic phase and solution-state NMR "invisible" molecules forming the rigid fibres of supramolecular hydrogels.[2] The observed line broadening results from the anisotropy imposed to the gelator molecules by their incorporation in partially mobile structures. For these Phe-based hydrogels, 40-50 % of gelator molecules are structural components of the fibres.

The calculation of fraction of ^{1}H peak intensity and variation of full width at half maximum are highly dependent on the time of the acquisition of the initial spectra and on the kinetics of gelation (which can be affected by sample volume and temperature of the NMR tube).

Table S6. Fraction of ^{1}H peak intensity ($\int_{gel}$) of spectra acquired 24 h after quenching hot solutions of Phe, Phe/Leu (5:1), Phe/Ser (5:1), Phe/Trp (5:1) and Phe/Tyr (5:1) (303 mM), in comparison with the ^{1}H peak intensity of spectra acquired immediately (performed at 298 K).

			$\int_{gel}$ / %					
	**Phe Arom**	**Phe $C_\alpha H$**	**Phe $C_\beta H_2$**	**Additive molecule**				
**Phe**	61	59	60	-	-	-	-	-
**Phe/Leu (5:1)**	50	51	49	Leu $C_\alpha H$ 103	Leu CH+$C_\beta H_2$ 98	Leu $(CH_3)_2$ 99	-	-
**Phe/Ser (5:1)**	68	80[a]	67	Ser $C_\alpha H$ 98	Ser $C_\beta H_2$ 80[a]	-	-	-
**Phe/Trp (5:1)**	56	58[b]	53	Trp H_4 88[c]	Trp H_7 115[c]	Trp H_5 81	Trp $C_\alpha H$ 58[b]	Trp $C_\beta H_2$ 80
**Phe/Tyr (5:1)**	56	56	55	Tyr Arom 61	-	-	-	-

[a] Overlapped

[b] Overlapped

[c] Broad baseline due to Phe Arom peak

Table S7. Variation of full width at half maximum (ΔFWHM) of ^{1}H-NMR spectra acquired immediately and 24 h after quenching hot solutions of Phe, Phe/Leu (5:1), Phe/Ser (5:1), Phe/Trp (5:1) and Phe/Tyr (5:1) (303 mM) (performed at 298 K).

			ΔFWHM / Hz					
	**Phe Arom**	**Phe $C_\alpha H$**	**Phe $C_\beta H_2$**	**Additive molecule**				
**Phe**	7.5	3.9	2.6	-	-	-	-	-
**Phe/Leu (5:1)**	13.4	9.3	7.6	Leu $C_\alpha H$ 1.1	Leu CH+$C_\beta H_2$ -8.0[a]	Leu $(CH_3)_2$ 1.5	-	-
**Phe/Ser (5:1)**	1.8	0.4[b]	0.0	Ser $C_\alpha H$ 0.1	Ser $C_\beta H_2$ 0.4[b]	-	-	-
**Phe/Trp (5:1)**	10.9	3.4[c]	4.7	Trp H_4 4.9	Trp H_7 5.2	Trp H_5 6.1	Trp $C_\alpha H$ 3.4[c]	Trp $C_\beta H_2$ 5.3
**Phe/Tyr (5:1)**	8.0	3.5	1.4	Tyr Arom 4.8	-	-	-	-

[a] Multiplet

[b] Overlapped

[c] Overlapped

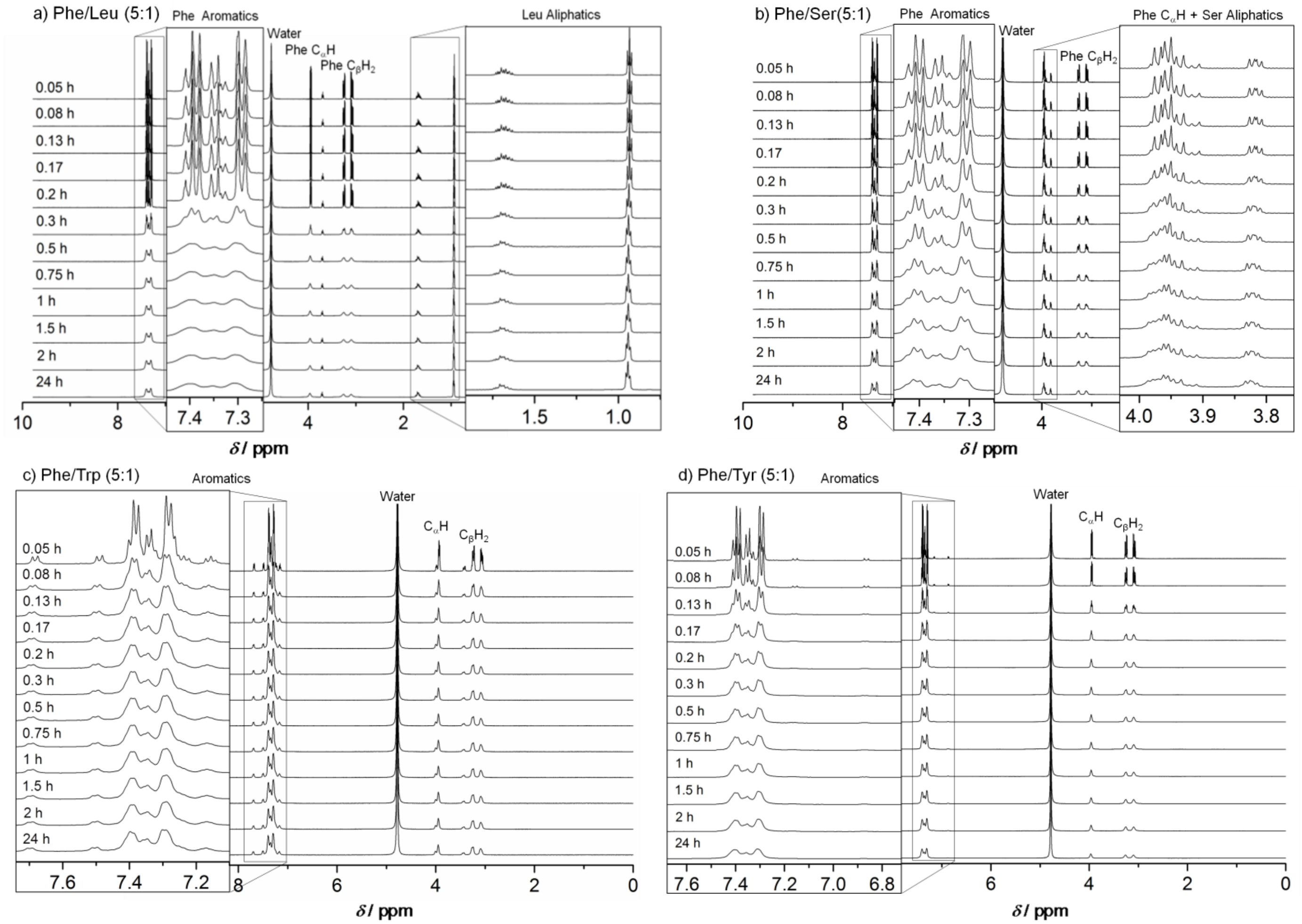

Figure S8. Kinetics of gelation monitored by the acquisition of ^{1}H solution-state NMR spectra over time, immediately after cooling down hot solutions of **a)** Phe/Leu (5:1), **b)** Phe/Ser (5:1), **c)** Phe/Trp (5:1) and **d)** Phe/Tyr (5:1) (performed at 298 K). Phe, Trp and Tyr peak ^{1}H become broader as a consequence of gelation, whereas Leu and Ser ^{1}H peaks remain sharp even after the hydrogel is formed.

8.3.1. ^{1}H-^{1}H 2D NOESY experiments

Negative nOe enhancements (blue) were detected in ^{1}H-^{1}H 2D NOESY NMR spectra for Phe protons in Phe-based hydrogels (Figure S9a-e). Negative cross-peaks are characteristic of large molecules which transfer magnetisation efficiently through dipolar interactions.[2] Since these Phe-based hydrogel systems are composed exclusively of LMW species, these findings indicated that molecules in solution contain information from the fibrous network due to their fast dynamics of exchange in the NMR frequency time scale.

Strong negative cross-peaks were also recorded between Phe and Trp or Tyr (Figure S9d and e), in hydrogels of Phe/Trp and Phe/Tyr, supporting that Trp and Tyr were in close proximity with Phe due to their incorporation in the hydrogel fibres.

The presence of weak negative cross-peaks between Phe and Leu (Figure S9b) indicated spatial proximity between both molecules. Despite the evidence for fast interaction of Leu with Phe at the gel/solution interfaces, the detection of positive spatial correlations between Leu protons (green), associated with small molecules, showed this molecule exists mainly in a free dissolved state. These findings were in agreement with the sharp Leu peaks (Figure S8) and the unmodified Leu peak integral (Table S6) observed for Leu after gelation.

No cross-peaks were observed between Phe and Ser in the hydrogel of Phe/Ser (Figure S9c).

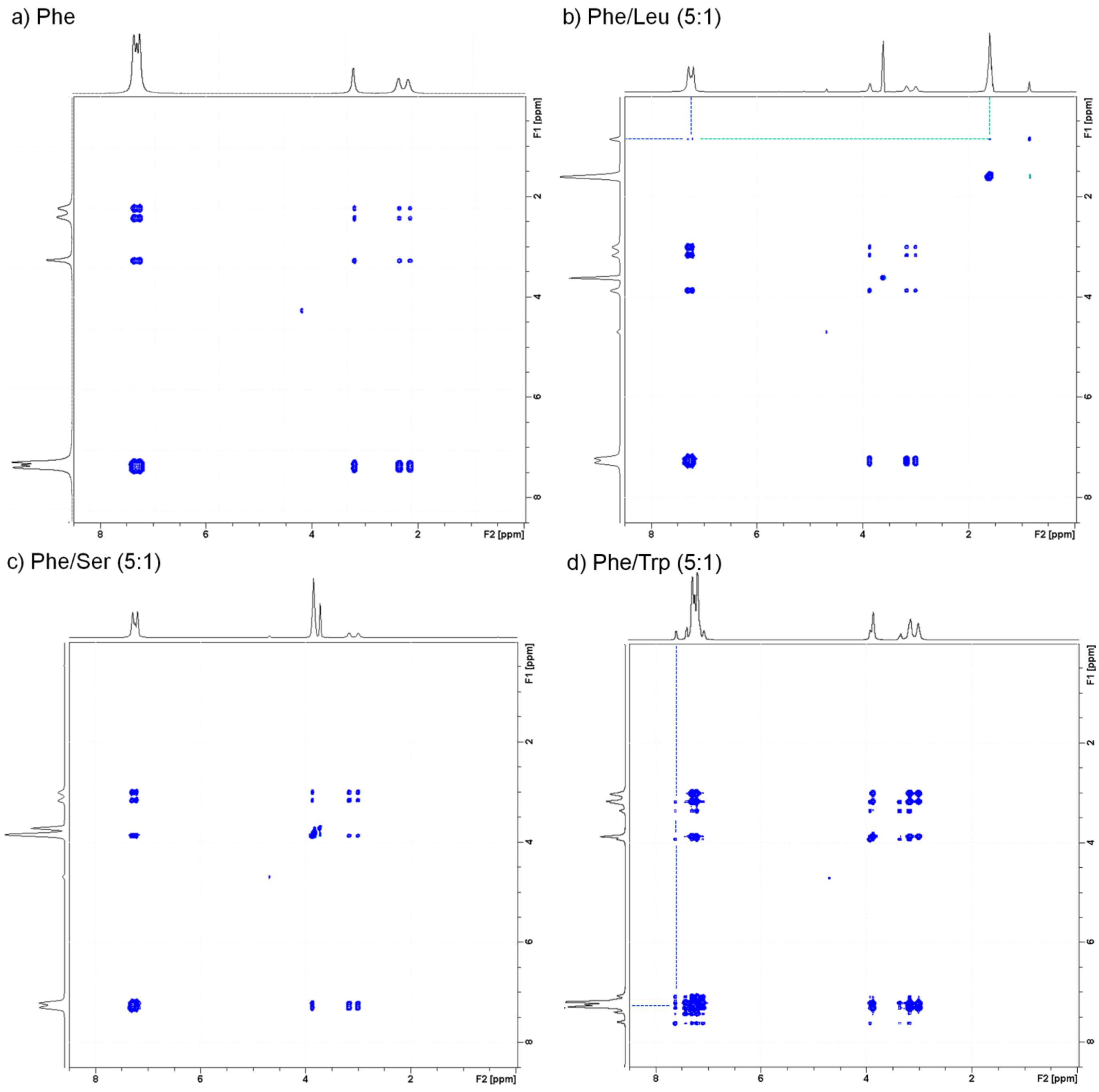

e) Phe/Tyr (5:1)

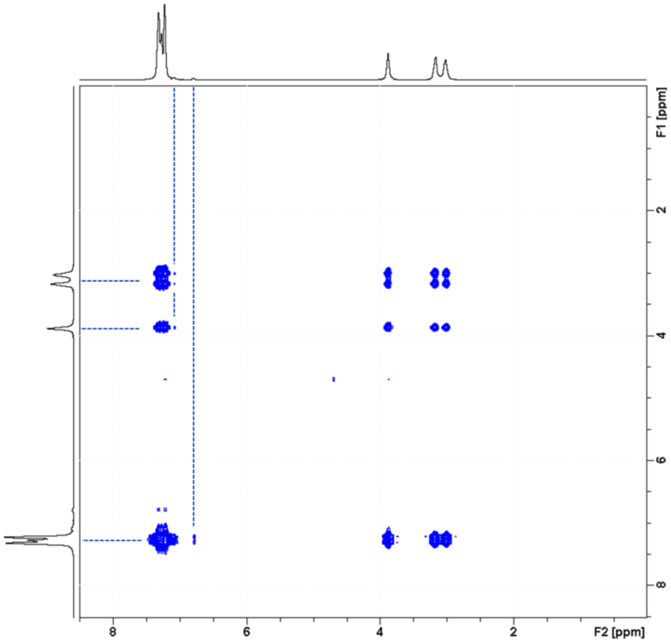

Figure S9. ^{1}H-^{1}H 2D NOESY spectra of hydrogels of Phe, Phe/Leu (5:1), Phe/Ser (5:1), Phe/Trp (5:1) and Phe/Tyr (5:1) acquired with a mixing time of 0.5 s. Negative nOe enhancements (blue), characteristic of large molecules, indicate that free gelator molecules contain properties from the fibrous network. Blue dashed lines highlight intermolecular correlations between Phe and **b)** Leu, **d)** Trp and **e)** Tyr, which are absent in hydrogels of Phe/Ser (**c**). Positive nOe enhancements (green), characteristic of small molecules, are highlighted by green dashed lines and correspond to correlations between Leu molecules in solution (**b**).

8.3.2. Longitudinal relaxation times experiments

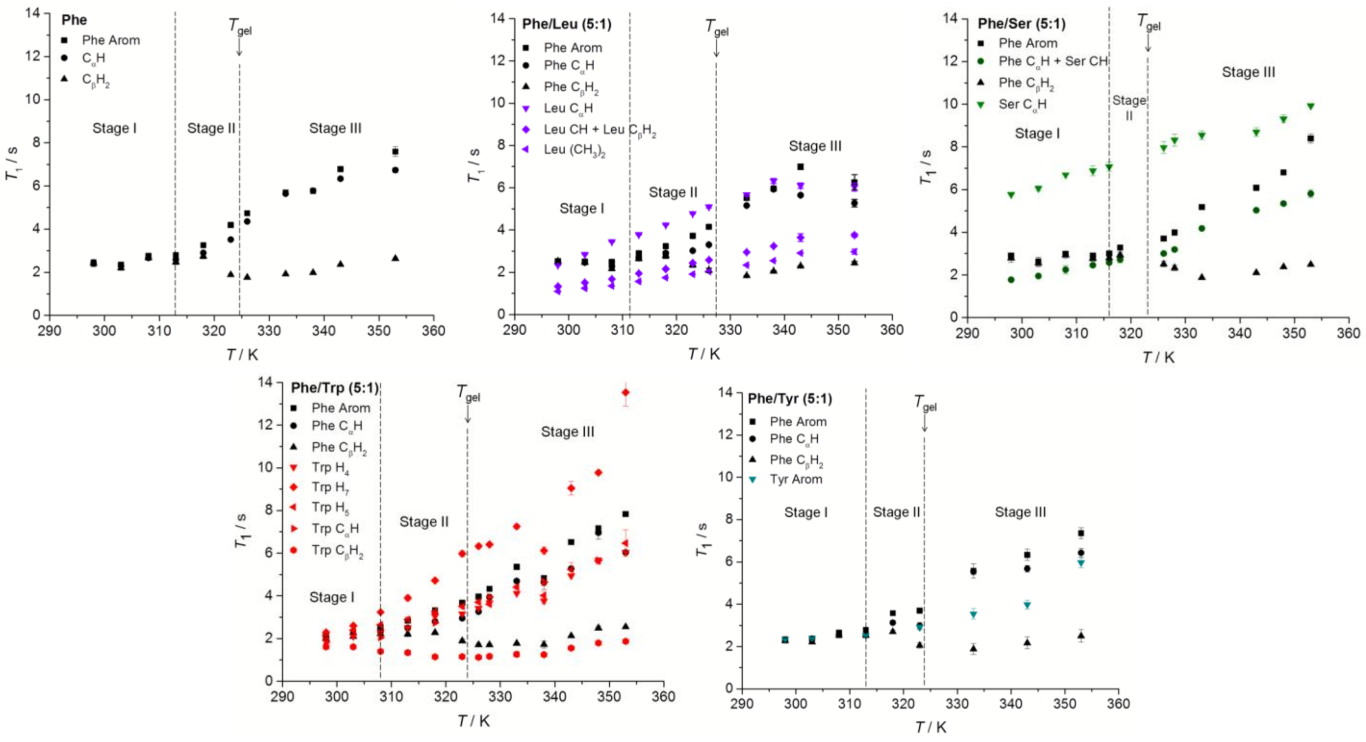

Figure S10. ^{1}H solution-state NMR T_1 times of Phe, Leu, Ser, Trp and Tyr in hydrogels of Phe, Phe/Leu (5:1), Phe/Ser (5:1), Phe/Trp (5:1) and Phe/Tyr (5:1), recorded from 298 to 353 K.

Table S8. ^{1}H solution-state NMR longitudinal relaxation times (T_1) for the hydrogels of Phe, Phe/Leu (5:1), Phe/Ser (5:1), Phe/Trp (5:1) and Phe/Tyr (5:1) measured at 298 K, with error values in parenthesis.

							**T_1 / s**		
	**Phe Arom**	**Phe C_αH**	**Phe $C_\beta H_2$**	**Water**			**Additive molecule**		
**Phe/Trp (5:1)**	2.13 (0.07)	2.04 (0.08)	2.00 (0.09)	12.99	Trp H4<br>1.97 (0.08)	Trp H7<br>2.29 (0.06)	Trp H5<br>2.15 (0.07)	Trp C_αH<br>1.83 (0.08)	Trp $C_\beta H_2$<br>1.61 (0.11)
**Phe/Tyr (5:1)**	2.34 (0.11)	2.29 (0.12)	2.28 (0.12)	12.05	Tyr Arom<br>2.34 (0.09)	-	-	-	-
**Phe**	2.45 (0.14)	2.42 (0.15)	2.41 (0.15)	11.31	-	-	-	-	-
**Phe/Leu (5:1)**	2.52 (0.12)	2.51 (0.13)	2.51 (0.12)	11.75	Leu C_αH<br>2.35 (0.02)	Leu<br>CH+$C_\beta H_2$<br>1.33 (0.03)	Leu $(CH_3)_2$<br>1.10 (0.02)	-	-
**Phe/Ser (5:1)**	2.91 (0.19)	1.78[a] (0.03)	2.78 (0.19)	12.07	Ser C_αH<br>5.78 (0.05)	Ser $C_\beta H_2$<br>1.78[a] (0.03)	-	-	-

[a] Represents an averaged value between Phe C_αH and Ser $C_\beta H_2$.

8.3.3. STD NMR experiments

STD NMR spectroscopy is applied frequently to identify the functional groups of a ligand responsible for binding to its receptor (a protein, typically).[3] This method relies on the transfer of saturation through cross-relaxation from a large saturated protein to a small bound ligand.[4] For amino acid based hydrogels, we can consider the network as the supramolecular entity that can be saturated selectively. Considering an analogous dependence of η_{STD} with concentration as in the case of protein-ligand studies:

$$\eta_{STD}=\frac{I_{STD}}{I_0}=\alpha_{STD}\frac{[PL]}{[L]_T}=\alpha_{STD}\frac{[Net\text{-}G]}{[G]_T}, \qquad \text{S1}$$

Where I_{STD} is the signal intensity from the difference spectrum, I_0 is the signal intensity from the STD_{off} spectrum and α_{STD} is a dimensionless scaling factor.[5] We considered that the concentration of the network-bound gelator, [Net-G], and the total gelator tion, $[G]_T$, were equivalent to the concentration of protein receptor-ligand complex, [PL], and the total ligand concentration, $[L]_T$, respectively.[5]

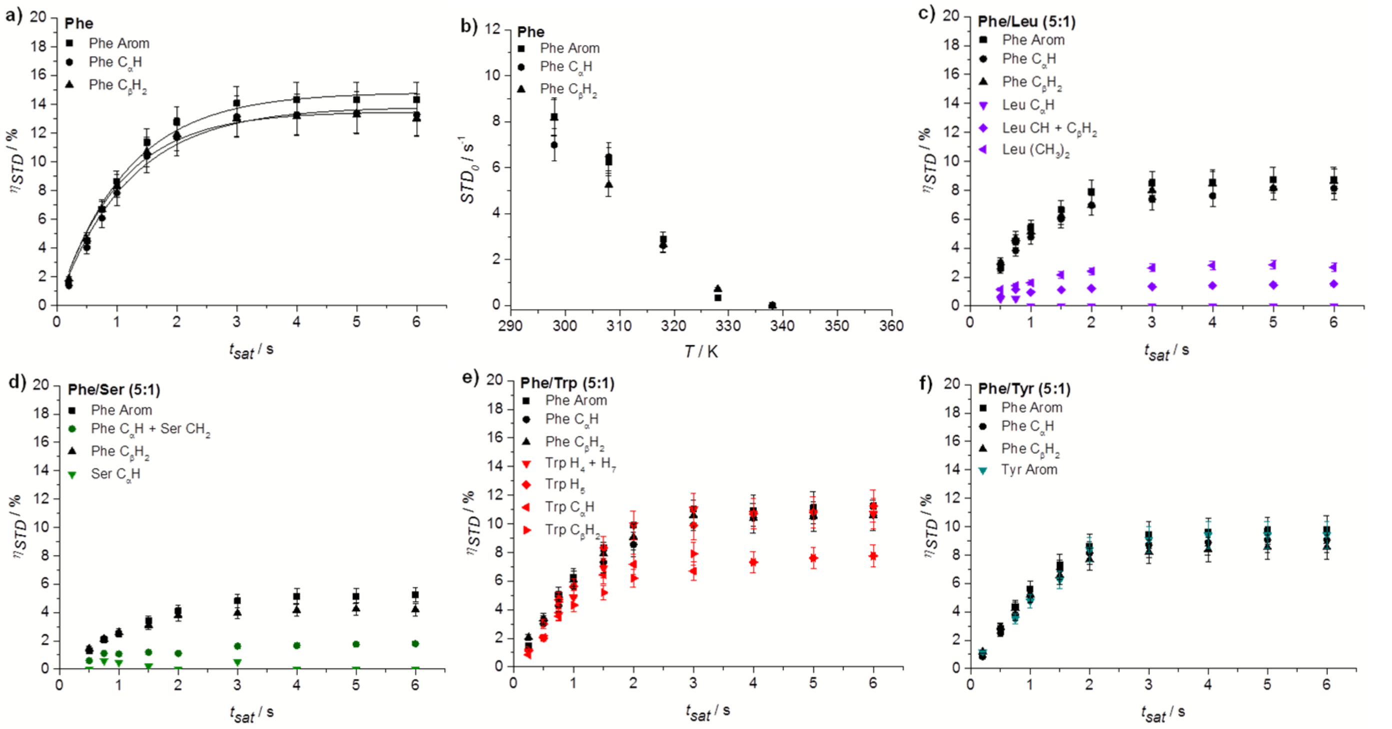

Figure S11. Build-up curves of η_{STD} in hydrogels of **a)** Phe, **c)** Phe/Leu (5:1), **d)** Phe/Ser (5:1), **e)** Phe/Trp (5:1) and **f)** Phe/Tyr (5:1) acquired at 298 K (STDon = 0 ppm and STDoff = 40 ppm). **b)** Initial slope values recorded from 298 to 338 K upon saturation of the network (STD_{on} = 0 ppm and STD_{off} = 40 ppm) in the hydrogel of Phe.

The STD parameters in these studies were reproducible with a mean error of 9.6 %.

Table S9. Initial slope values of fractional STD response (STD_0), average (x) and error (δ) values for the hydrogel of Phe measured at 298 K (STD_{on} = 0 ppm and STD_{off} = 40 ppm).

	1. STD_0 / s^{-1}	2. STD_0 / s^{-1}	3. STD_0 / s^{-1}	x	δ / %	xδ / %
**Phe Arom**	12.8	13.7	11.6	12.7	8.3	
**Phe $C_\alpha H$**	12.9	13.8	11.1	12.6	11.0	9.6
**Phe $C_\beta H_2$**	12.9	13.8	11.4	12.7	9.6	

Table S10. Initial slope values of fractional STD response (*STD_0*) for the hydrogels of Phe, Phe/Leu (5:1), Phe/Ser (5:1), Phe/Trp (5:1), Phe/Tyr (5:1) and Phe/Trp/Tyr (5:1:1) measured at 298 K (STD_{on} = 0 ppm and STD_{off} = 40 ppm).

	**Phe Arom**	**Phe $C_\alpha H$**	**Phe $C_\beta H_2$**	***STD_0* / s^{-1}** **Additive molecule**				
**Phe/Trp (5:1)**	8.26	7.77	7.93	Trp H4 7.25	Trp H7 7.25	Trp H5 7.72	Trp $C_\alpha H$ 7.41	Trp $C_\beta H_2$ 6.91
**Phe/Tyr (5:1)**	9.22	7.47	9.21	Tyr Arom 7.01	-	-	-	-
**Phe**	12.80	11.52	12.90	-	-	-	-	-
**Phe/Leu (5:1)**	8.14	7.03	7.93	Leu $C_\alpha H$ 0	Leu CH+$C_\beta H_2$ 1.86	Leu $(CH_3)_2$ 2.67	-	-
**Phe/Ser (5:1)**	3.54	1.53[a]	3.93	Ser CαH 0	Ser CβH2 1.53[a]	-	-	-

[a] Represents an averaged value between Phe $C_\alpha H$ and Ser $C_\beta H_2$.

[1] B. Khawas, G. Krishna Murti, Acta Crystallographica Section B: Structural Crystallography and Crystal Chemistry **1969**, 25, 1006-1009.

[2] S. Bouguet-Bonnet, M. Yemloul, D. Canet, Journal of the American Chemical Society **2012**, 134, 10621-10627.

[3] M. Mayer, B. Meyer, Angewandte Chemie International Edition **1999**, 38, 1784-1788.

[4] J. Angulo, P. M. Nieto, European Biophysics Journal **2011**, 40, 1357-1369.

[5] C. A. Lepre, J. M. Moore, J. W. Peng, Chemical reviews **2004**, 104, 3641-3676.
